# Variation in defensive and exploratory behaviors across a rattlesnake (*Crotalus scutulatus* × *viridis*) hybrid zone in southwestern New Mexico

**DOI:** 10.1038/s41598-025-96155-8

**Published:** 2025-04-08

**Authors:** Dylan W. Maag, Yannick Z. Francioli, Matthew T. H. Goetz, Lea N. Sanders, Xochitl Lopez, Todd A. Castoe, Gordon W. Schuett, Rulon W. Clark

**Affiliations:** 1 Department of Evolution, Ecology, and Organismal Biology, University of California, Riverside, Riverside, CA USA; 2 Department of Life, Earth, and Environmental Sciences, West Texas a&M University, Canyon, TX USA; 3 Department of Biology, University of Texas at Arlington, Arlington, TX USA; 4 Department of Biology, San Diego State University, San Diego, CA USA; 5 Department of Biology | Neuroscience Institute, Georgia State University, Atlanta, GA USA; 6 Chiricahua Desert Museum, Rodeo, NM USA; 7 WTAMU, Natural Sciences Building 329, Canyon, TX 79016 USA

**Keywords:** Ecology, Behavioural ecology, Evolutionary ecology, Behavioural genetics, Evolutionary biology, Genetic hybridization, Zoology, Animal behaviour, Herpetology

## Abstract

Studies on animal temperaments (consistent differences in behaviors across contexts) and behavioral syndromes (suites of correlated behaviors across contexts) have surged in recent decades. Accordingly, behavioral ecologists have gained greater appreciation for their evolutionary role and significance. Yet, despite their importance as potential evolutionary drivers, research focused on temperament and syndromes in shaping hybridization events is vastly understudied. Case studies have shown that hybridization has multiple effects on these phenomena, such as eliminating syndromes present in parental lineages and generating novel syndromes within hybrids. Here, we assessed temperament and syndromes in a naturally occurring rattlesnake hybrid zone (*Crotalus scutulatus* × *viridis*). We used laboratory behavioral assays to quantify defensive and explorative behaviors, and tested whether these traits were correlated with spatial and hunting behaviors of free-ranging individuals. *C. viridis* was more significantly more prone to rattle than *C. scutulatus* during handling tests. Similarly, hybrid individuals that had a greater proportion of their genome derived from *C. viridis* were also more prone to rattle. Parental and hybrid snakes exhibited varying syndromes in defensiveness and exploratory behaviors, yet further research is necessary to determine whether they impact hybrid fitness by creating mismatches between temperaments and predation pressures under natural conditions.

**Supplementary Information:**

The online version contains supplementary material available at 10.1038/s41598-025-96155-8.

## Introduction

Behavior has long been recognized as both a highly variable and evolutionarily labile aspect of an organism’s phenotype, and an increasing number of studies in recent decades document consistent patterns of behaviors (i.e., temperament) expressed by individuals across different contexts^1,2^. Temperament (also referred to in the literature as “animal personality”) is typically measured on binary (e.g., bold vs. shy, explorative vs. non-explorative, aggressive vs. submissive) or continuous scales. For example, individuals that are on the bolder side of the bold/shy continuum are predicted to prioritize high risk/high reward behaviors consistently across different contexts (e.g., time, age, reproductive status, foraging, and social interactions). Temperaments appear to be moderately heritable and thus shaped by both environmental and genetic variation^3^. Behavioral ecologists also make a distinction between temperament and behavioral syndromes, which are defined by the correlation between two or more behavioral temperaments expressed by an individual across time or context^4,5^.

Although behavioral variation associated with temperament and behavioral syndromes (henceforth termed syndromes) has been studied widely across taxa and in a number of ecological and evolutionary contexts^1,3,6–11^, our understanding of these phenomena in the context of interspecific hybridization is understudied. In hybrid populations, for example, genetic and phenotypic variability is often higher between individuals owing to interspecific gene flow from different parental lineages^12^. This type of admixture could theoretically lead to the breakdown of suites of correlated traits—which may lead to hybrid inferiority when co-adapted traits optimize fitness^13^. Accordingly, syndromes might collapse across hybrid zones and, in turn, represent an extrinsic barrier to further hybridization, assuming their collapse leads to non-optimal expression of behaviors in critical environmental contexts^14^. Conversely, selection can also have a role in strengthening syndromes in hybrids^15^, but there is mixed support for this outcome.

Hybrids between benthic and planktivorous morphs of Arctic Charr (*Salvelinus alpinus*) have been shown to express repeatability in boldness and sociability, but to a lesser degree than the parental individuals. In this case, no evidence for the relaxation of behavioral syndromes in hybrids was found^14^. However, other studies have found that syndromes in hybrids tend to be weaker than in parentals. Parental salmon morphs (*Salmo salar*) expressed distinct syndromes associated with boldness, aggression, and exploration, whereas hybrids only displayed a syndrome between boldness and response to a novel object^16^. Similarly, hybrids of two pufferfish species (*Takifugu rubripes* and *T. niphobles*) had similar, but statistically weaker, syndromes than the parentals in a variety of temperament traits, including boldness and feeding responses^17^. Finally, hybrids can express unique and/or stronger syndromes than parentals. Hybrid swordtails (*Xiphophorus *spp.) showed a significant syndrome between boldness and defensiveness that was absent in the parental populations^15^. This situation is akin to the expression of transgressive or novel traits seen in many hybrid zones^18–20^, which can lead to adaptive evolution through transgressive segregation in the hybrids^21^. Though research is limited, temperament can act as a post-zygotic barrier if certain temperaments that are over- or under-expressed in hybrids are favored by natural selection in the habitat where hybridization occurs. Interestingly, a recent review specifically highlighted the potential for temperament to drive reproductive isolation^22^.

Sexual selection can favor either assortative or disassortative mating based on individual temperaments. In turn, this can shape pre-zygotic isolating mechanisms between species. For example, temperaments can shape spatial behaviors and habitat selection in ways that increase or decrease isolation between lineages, depending on context^22^. Additionally, a large number of studies have demonstrated how variation related to temperaments and syndromes shapes traits related to reproductive isolation, including variation in general activity levels^23–26^, exploration patterns^24,25,27,28^, dispersal^29,30^, foraging activity^7,26,31^, spatial behaviors^32^, anti-predator behaviors^25,33–36^, and reproductive success^7,8,27,37,38^.

Unquestionably, the literature on animal temperament is taxonomically uneven, with relatively few studies of non-avian reptiles^1,39^. Only two previous studies have examined temperament in pitviper snakes (Viperidae: Crotalinae). These studies found that rattlesnakes (Southern Pacific Rattlesnakes, *C. oreganus*, and Western Diamond-backed Rattlesnakes, *C. atrox*, respectively) exhibited quantifiable behavioral traits that were consistent within individuals while also showing syndromes in *C. atrox *with respect to movement activity, boldness, and sociability^40,41^ Because signatures of interspecific hybridization have been found repeatedly in *C. scutulatus*^42^, *C. viridis* and *C. oreganus*^43,44^ clades, these are ideal taxa in which to study the role of temperament and syndromes in shaping hybridization dynamics.

Here, we studied temperament and syndromes in rattlesnakes from a narrow band (12 km) of transitional/mosaic habitat region between the Chihuahuan and Sonoran deserts in southwestern New Mexico where Mojave Rattlesnakes (*Crotalus scutulatus*) and Prairie Rattlesnakes (*C. viridis*) hybridize. Specifically, we tested a large cohort of parental and hybrid individuals to quantify behavioral traits related to defensiveness and exploration. Furthermore, we assessed potential syndromes between temperament traits expressed in laboratory assays and both spatial behaviors^45^and hunting behaviors^46^ in free-ranging snakes to assess whether there are correlations between behaviors expressed in a captive context and the more ecologically relevant (but logistically challenging to acquire) field behaviors. Based on the potential for hybrids to exhibit increased variation in temperaments, we hypothesized that hybrids would be transgressive or intermediate, depending on the relative expression of temperament traits in parental species. We also hypothesized that parental populations would exhibit syndromes across defensive, exploratory, and spatial/hunting contexts, but that these syndromes would be weakly expressed (or absent) in hybrids.

## Results

### Behavioral assays

**Fig. 1:**
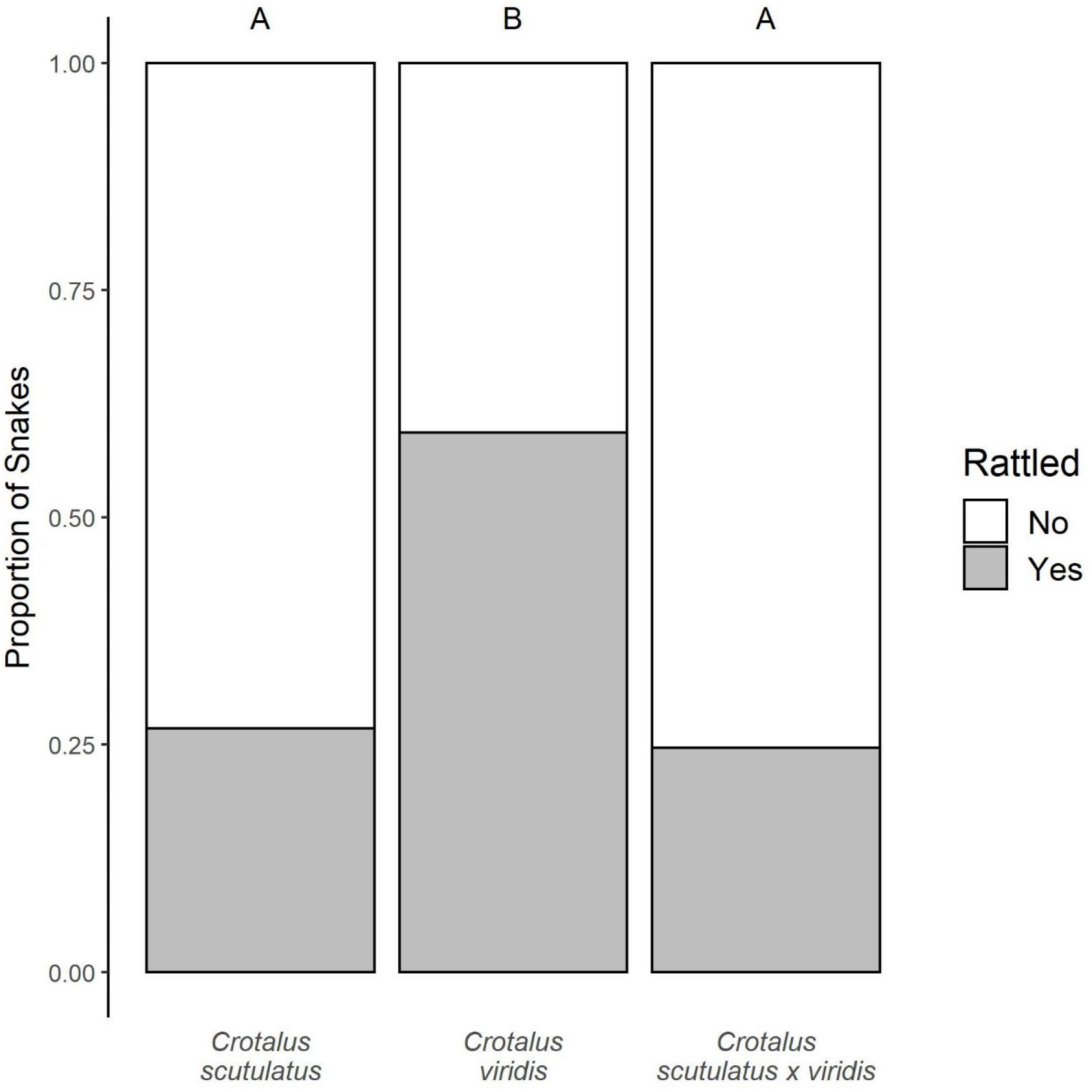
Bar graph of the proportions of snakes that rattled during the handling assay. Propensity to rattle was significantly different across groups (X^2^ = 20.8; df = 2; *p* < 0.001). More *Crotalus viridis* rattled than either *C. scutulatus* or *C. scutulatus* × *viridis* (post-hoc Tukey: z-ratio = −3.52, 3.98; *p* = 0.001, < 0.001; respectively), whereas there was no difference between *C. scutulatus* and *C. scutulatus* × *viridis* (post-hoc Tukey: z-ratio = −0.247, *p* = 0.967). Letters above the bars indicate statistically significant groupings of the genetic groups. Sample sizes: *C. scutulatus* = 41, *C. viridis* = 59, *C. scutulatus* × *viridis* = 85.

**Fig. 2:**
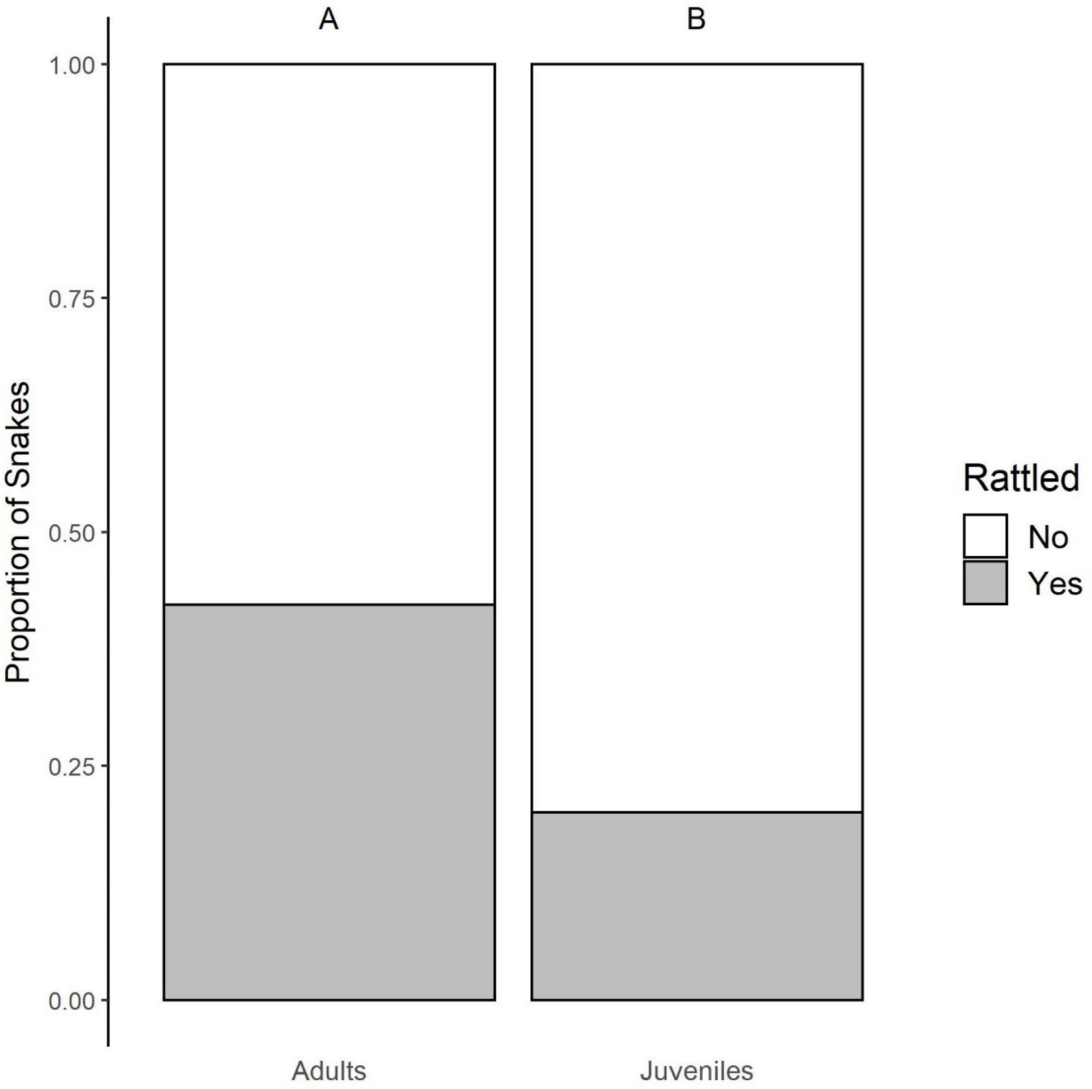
Bar graph of the proportions of adult versus juvenile snakes that rattled during the handling assay (X^2^ = 9.38; df = 1; *p* = 0.002). Letters above the bars indicate statistically significant groupings of the genetic groups. Sample sizes: adults = 135, juveniles = 50.

We conducted behavioral assays on 185 rattlesnakes: 41 Mojave Rattlesnakes (*Crotalus scutulatus*; adults = 36; juveniles = 5; male = 30; non-pregnant females = 11), 59 Prairie Rattlesnakes (*C. viridis*; adults = 43; juveniles = 16; male = 43; non-pregnant females = 16), and 85 hybrid rattlesnakes (*C. scutulatus* × *viridis*; adults = 56; juveniles = 29; male = 53; non-pregnant females = 32). Pregnant females were not included in this study due to the lack of sample size (*C. scutulatus* = 1, *C. viridis* = 3, and *C. scutulatus* × *viridis* = 6). The most informative models for explaining variation in rattle behavior were those containing genetic group + age and genetic group * age. Therefore, we report the results from the model containing genetic group + age as the two predictor variables. We found both genetic group (X^2^ = 20.8, df = 2, *p* < 0.001) and age (X^2^ = 9.38, df = 1, *p* = 0.002) were significant predictors of rattling behavior. *Crotalus scutulatus* rattled less frequently (26.8% of individuals rattled defensively) than *C. viridis* (59.3%; post-hoc Tukey: z-ratio = −3.52, *p* = 0.001), but were not significantly different than the hybrids (25.6%; post-hoc Tukey: z-ratio = −0.247, *p* = 0.967). Hybrid subjects also rattled significantly less frequently than *C. viridis* (post-hoc Tukey: z-ratio = 3.98, *p* < 0.001; Fig. 1). Overall, adult snakes of all categories were more likely to rattle (42.2% rattled) than juveniles (20% rattled; Fig. 2).

During exploratory assays, snakes spent an average of 8.17 min in hideboxes (13.6% of the total time), but this was highly variable (Table 1). For time spent in hidebox, the model with genetic group as the sole fixed factor was the most informative for analysis. However, the relationship between group and time in hidebox was not significant (X^2^ = 0.231; df = 2, 181; *p* = 0.891). Groups also did not differ in the variability of this behavior (F = 0.392; df = 2, 181; *p* = 0.677).

**Table 1:** Exploratory behaviors assayed during the open-field test. Snakes in different groups did not exhibit significant differences in behaviors. Time spent in a hidebox and motionless are reported as the proportion of time during the 60-minute assay snakes displayed these behaviors.

Exploratory behavior	Crotalus scutulatus *(n = 40)*	Crotalus viridis *(n = 59)*	Crotalus scutulatus × viridis *(n = 85)*	X^2^	*P*-value
Time spent in a hidebox	0.169 ± 0.052	0.117 ± 0.034	0.133 ± 0.031	0.231	0.891
Time spent motionless	0.537 ± 0.059	0.619 ± 0.045	0.572 ± 0.036	0.692	0.708
Number of quadrant transitions	7.900 ± 1.820	5.590 ± 1.190	6.070 ± 0.990	1.330	0.513

For the number of quadrant transitions, the most informative model contained only genetic group as the predictor variable and no significance was found between number of quadrant transitions and genetic group (X^2^ = 2.05, df = 2,181, *p* = 0.360). Snakes performed an average of six quadrant transitions during the 60 min exploratory assay, but were equally variable within and between groups (F = 0.773; df = 2, 181; *p* = 0.463; Table 1).

For time spent motionless during open field tests, the most informative models contained genetic group and genetic group + age as the fixed factors, and so we report the results of the model with only genetic group. Again, we found no difference between the groups in the amount of time that they spent motionless (X^2^ = 0.692; df = 2, 181; *p* = 0.708). Snakes spent an average of 34.7 min (57.9%) motionless outside of a hidebox. The variability of this trait was also high, but not significantly different between the groups (F = 0.867; df = 2, 181; *p* = 0.422; Table 1).

For individuals tested in the threat assay (*C. scutulatus* = 32, *C. viridis* = 47, *C. scutulatus* × *viridis* = 55), the model containing genetic group was the most informative. Overall, only 24.6% of the snakes struck defensively and we found no relationship between the number of snakes that struck during the assay and their genetic group (X^2^ = 3.26, df = 2, *p* = 0.196; Figure S1).

### Behavior and hybrid index

**Fig. 3:**
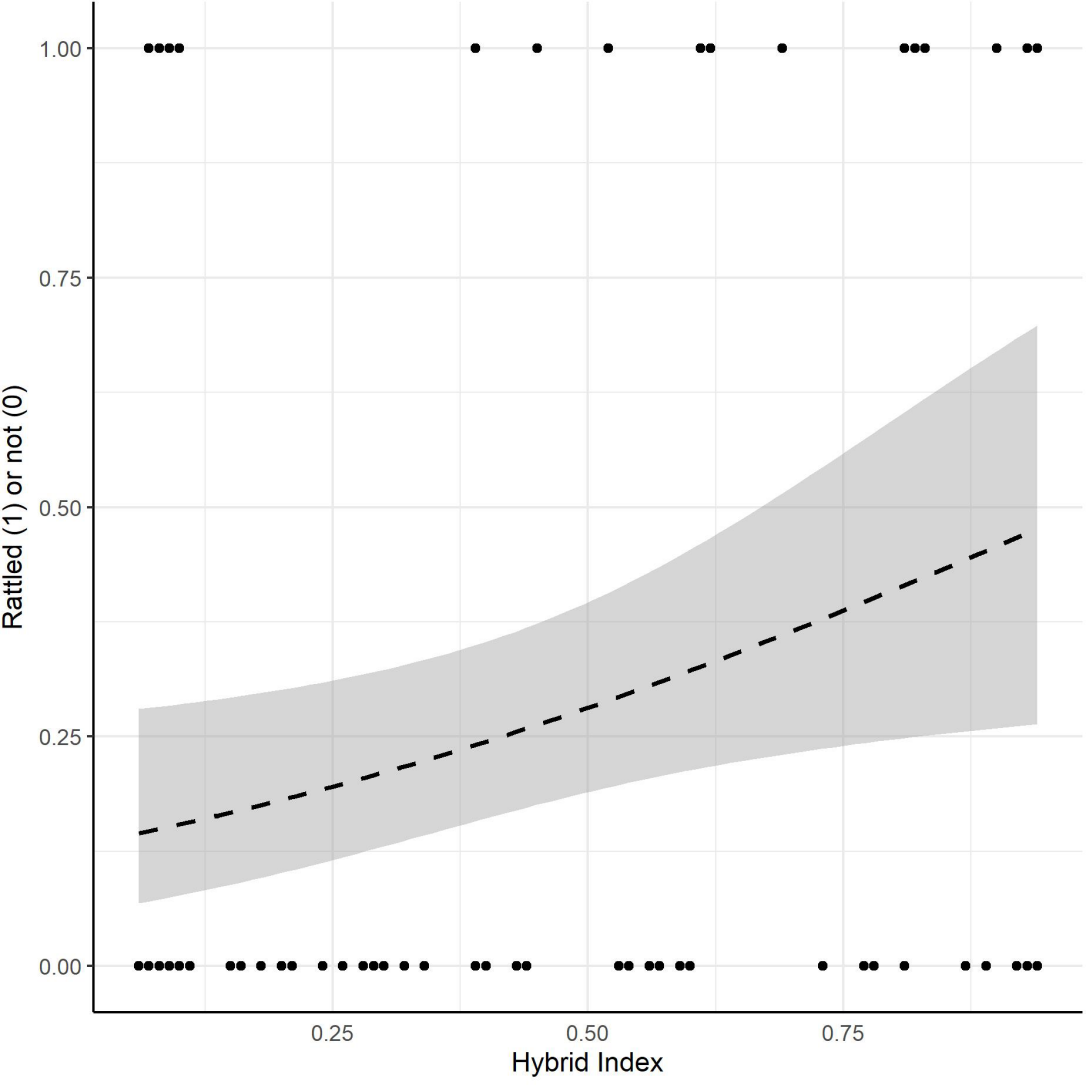
Scatter plot of propensity to rattle versus hybrid index (proportion of genome derived from *C. viridis*) for hybrid snakes. The dotted line is the binomial regression line (X^2^ = 5.45, df = 1, *p* = 0.020) and the shaded region flanking either side of the line is ± 1 SE. Sample size = 85.

For our analysis of hybrid index (HI—proportion of the genome derived from *C. viridis*) and rattling behavior, the most informative models contained HI and HI + age. Hence, we report the results of the model containing HI as the only fixed factor. Individuals with higher HIs (i.e., more *C. viridis*-like) were significantly more likely to rattle during the handling assay (X^2^ = 5.45, df = 1, *p* = 0.020; Fig. 3).

The most informative models for time in a hidebox contained HI, HI + age, and HI * age, time spent motionless contained HI, HI + age, HI * age, and HI * age + sex, and number of quadrant transitions contained HI * age, HI * age + sex as the predictor variables. Therefore, we report the results of the model containing HI as the only predictor variable for the time a hybrid snake spent in a hidebox and motionless and the results of the model containing HI * age for the number of quadrant transactions snakes performed. None of the exploratory behaviors had a significant relationship with the hybrid index (time spent in a hidebox [X^2^ = 0.417, df = 1, *p* = 0.519], number of quadrant transitions [X^2^ = 0.138, df = 1, *p* = 0.710], and time spent motionless [X^2^ = 0.585, df = 1, *p* = 0.445]). We initially found that the interaction between age and HI of the snakes significantly affected the number of quadrant transitions (X^2^ = 7.78, df = 1, *p* = 0.005), but visualization of this relationship resulted in identification of two statistical outliers in the juvenile sample (using the Minimum Covariance Determinant method since the data was heavily skewed); when reanalyzing the model without these two outliers the number of quadrant transitions was not significantly related to the age of hybrid snakes (X^2^ = 3.513, df = 1, *p* = 0.061) and the interaction between age and HI of the snakes was not significant (X^2^ = 3.232, df = 1, *p* = 0.072). A plot of this relationship is presented in Figure S.2. The most informative model for striking during the threat assay contained only HI. We did not find a significant relationship between hybrid index and striking (X^2^ = 0.190, df = 1, *p* = 0.663).

### Behavioral syndromes: defensiveness vs. exploration

We found that the number of behavioral syndromes expressed among individuals differed between groups. Adult *C. scutulatus* displayed behavioral syndromes between defensive striking (i.e., striking during the threat assay) and time spent motionless (X^2^ = 3.99, df = 1, *p* = 0.046, Table 2) and quadrant transitions (X^2^ = 7.85, df = 1, *p* = 0.005, Table 2). *Crotalus scutulatus* that spent more time motionless were more likely to strike during the threat assay, and *C. scutulatus* that performed more quadrant transitions were also less likely to strike during the threat assay. *Crotalus viridis* exhibited significant relationships between time in a hidebox and defensive striking (X^2^ = 6.21, df = 1, *p* = 0.013, Table 2), time spent motionless and defensive striking (X^2^ = 6.71, df = 1, *p* = 0.010, Table 2), and quadrant transitions and defensive striking (X^2^ = 5.80, df = 1, *p* = 0.016, Table 2). Adult *C. viridis* who spent more time within a hidebox or motionless were less likely to strike during the threat assay. However, like *C. scutulatus*, *C. viridis* that performed more quadrant transitions were also less likely to strike during the threat assay. We found two behavioral syndromes in *C. scutulatus* × *viridis*: adult hybrids that spent more time motionless or performed more quadrant transitions, were less likely to rattle during the handling assay (X^2^ = 6.22, 9.28; df = 1, 1; *p* = 0.013, 0.002; Table 2). We did not find any evidence of behavioral syndromes in juvenile subjects of *C. viridis* or *C. scutulatus* × *viridis*. Owing to small sample size (*n* = 5), we were not able to analyze behavioral syndromes of juvenile *C. scutulatus*.

**Table 2:** Behavioral syndromes between defensiveness (handling and threat assays) and exploration (open-field test). Boldened rows signify the existence of a significant behavioral syndrome identified with binomial generalized linear model approach. Arrows indicate directionality of relationship between defensiveness and explorativeness. TH = Time in a hidebox. TM = Time spent motionless. QT = Quadrant transitions.

Defensive behavior	Exploratory behavior	X^2^	*P*-value	Syndrome relationship	
				Defensiveness	Explorativeness
*Crotalus scutulatus* *(n = 40)*					
Rattling (Adults)	TH	1.86	0.173	–	–
	TM	2.40	0.121	–	–
	QT	0.660	0.417	–	–
Striking	TH	3.24	0.072	–	–
	**TM**	**3.99**	**0.046**	↑	↓
	**QT**	**7.85**	**0.005**	↑	↓
*Crotalus viridis* *(n = 59)*					
Rattling (Adults)	TH	0.032	0.858	–	–
	TM	0.086	0.770	–	–
	QT	3.22	0.073	–	–
Rattling (Juveniles)	TH	2.06	0.151	–	–
	TM	2.49	0.115	–	–
	QT	2.65	0.103	–	–
Striking	**TH**	**6.21**	**0.013**	↓	↓
	**TM**	**6.71**	**0.010**	↓	↓
	**QT**	**5.80**	**0.016**	↓	↓
*Crotalus scutulatus* × *viridis* *(n = 85)*					
Rattling (Adults)	TH	0.425	0.514	–	–
	**TM**	**6.22**	**0.013**	↓	↓
	**QT**	**9.28**	**0.002**	↓	↑
Rattling (Juveniles)	TH	0.566	0.452	–	–
	TM	1.03	0.309	–	–
	QT	2.60	0.107	–	–
Striking	TH	0.008	0.929	–	–
	TM	0.383	0.536	–	–
	QT	0.362	0.548	–	–

### Behavioral syndromes: exploration vs. field behaviors

**Fig. 4:**
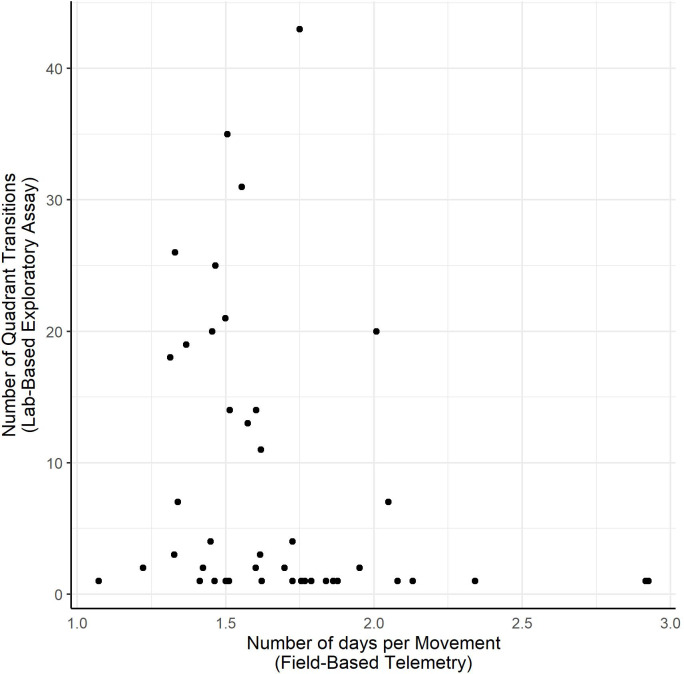
Scatter plot showing the negative correlation (behavioral syndrome) between the number of days per movement for individuals when they were free-ranging and the number of quadrant transitions those individuals performed during the exploratory assay. (Spearman correlation, Holm’s adjustment: *r* = 0.−386, *n* = 43, *p* = 0.032).

We found no evidence for behavioral syndromes within any of the groups between spatial or hunting behaviors measured in the field and exploratory behaviors assayed in the laboratory (Tables 3 and 4). After combining data from all groups, we found a moderately strong syndrome (*r* = − 0.386, *p* = 0.032) between spatial behavior (number of days per movement) and exploratory behavior, wherein snakes that moved more often in the field also tended to transition between quadrants more often during the exploratory assay (Fig. 4).

**Table 3:** Behavioral syndromes between exploratory behaviors measured in the open-field test and Spatial behaviors of free-ranging snakes^45^. Boldened rows signify the existence of a behavioral syndrome by way of spearman correlation with Holm’s adjusted p-values to account for multiple tests. 50% bbkde = the number of 50% isopleths estimated from their GPS locations by way of a brownian Bridge kernel density estimator (as an estimate for how patchy the core space use of the snakes). DMD = Distance the snakes moved on average per day (m/day). FM = The number of days between movements. NA = not applicable.

Lab-based exploratory behavior	Field spatial behaviors	*R*	Sample size	*P*-value
*Crotalus scutulatus*				
Time spent in a hidebox	50% bbKDE	0.278	13	0.847
	DMD	− 0.169	17	0.847
	FM	0.277	17	0.847
Number of quadrant transitions	50% bbKDE	0.150	13	1
	DMD	0.162	17	1
	FM	− 0.302	17	0.718
Time spent motionless	50% bbKDE	− 0.513	13	0.219
	DMD	0.015	17	1
	FM	0.009	17	1
*Crotalus viridis*				
Time spent in a hidebox	50% bbKDE	− 0.189	13	1
	DMD	0.186	15	1
	FM	− 0.247	15	1
Number of quadrant transitions	50% bbKDE	− 0.433	13	0.418
	DMD	0.361	15	0.418
	FM	− 0.227	15	0.418
Time spent motionless	50% bbKDE	0.263	13	0.850
	DMD	− 0.261	15	0.850
	FM	0.296	15	0.850
*Crotalus scutulatus* × *viridis*				
Time spent in a hidebox	50% bbKDE	NA	7	NA
	DMD	0.400	11	0.446
	FM	− 0.200	11	0.555
Number of quadrant transitions	50% bbKDE	0.116	7	1
	DMD	0.175	11	1
	FM	− 0.280	11	1
Time spent motionless	50% bbKDE	− 0.116	7	0.805
	DMD	− 0.418	11	0.539
	FM	0.436	11	0.539
All Groups				
Time spent in a hidebox	50% bbKDE	− 0.125	33	1
	DMD	0.112	43	1
	FM	− 0.012	43	1
Number of planes crossed	50% bbKDE	0.076	33	0.673
	DMD	0.227	43	0.288
	**FM**	**− 0.386**	**43**	**0.032**
Time spent motionless	50% bbKDE	− 0.081	33	0.656
	DMD	− 0.215	43	0.332
	FM	0.306	43	0.138

**Table 4:** Behavioral syndromes between exploratory behaviors measured in the open-field test and hunting behaviors expressed by free-ranging snakes^46^. Boldened p-values signify the existence of a behavioral syndrome by way of spearman correlation with Holm’s adjusted p-values to account for multiple tests. NPR = Nightly probing rate (probes/min). PE = Prey encounter frequency (prey/min). AT = Abandonment time. HF = Hunting frequency (number of nights tracked hunting/number of nights tracked). NA = not applicable.

Lab-Based Exploratory Behaviors	Field Hunting Behaviors	*R*	Sample Size	*P*-value
*Crotalus scutulatus*				
Time spent in a hidebox	NPR	− 0.082	16	1
	PE	− 0.158	16	1
	AT	0.113	15	1
	HF	− 0.069	20	1
Number of quadrant transitions	NPR	0.355	16	0.710
	PE	− 0.028	16	1
	AT	0.153	15	1
	HF	0.156	20	1
Time spent motionless	NPR	− 0.424	16	0.306
	PE	0.504	16	0.185
	AT	− 0.243	15	0.766
	HF	0.108	20	0.766
*Crotalus viridis*				
Time spent in a hidebox	NPR	− 0.602	14	0.091
	PE	− 0.232	13	0.892
	AT	0.462	14	0.290
	HF	0.107	16	0.892
Number of quadrant transitions	NPR	− 0.021	14	1
	PE	− 0.611	13	0.105
	AT	0.085	14	1
	HF	0144	16	1
Time spent motionless	NPR	0.380	14	0.540
	PE	0.580	13	0.150
	AT	− 0.174	14	1
	HF	− 0.062	16	1
*Crotalus scutulatus* × *viridis*				
Time spent in a hidebox	NPR	NA	10	NA
	PE	NA	10	NA
	AT	NA	10	NA
	HF	− −0.465	17	0.060
Number of quadrant transitions	NPR	0.276	10	0.880
	PE	0.387	10	0.810
	AT	−0.080	10	0.880
	HF	0.427	17	0.349
Time spent motionless	NPR	− 0.467	10	0.696
	PE	− 0.358	10	0.931
	AT	0.248	10	0.978
	HF	0.067	17	0.978
All Groups				
Time spent in a hidebox	NPR	0.010	40	1
	PE	− 0.135	39	1
	AT	− 0.062	39	1
	HF	− 0.098	53	1
Number of planes crossed	NPR	0.272	40	0.360
	PE	− 0.100	39	1
	AT	0.066	39	1
	HF	0.161	53	0.749
Time spent motionless	NPR	− 0.298	40	0.227
	PE	0.308	39	0.227
	AT	0.041	39	1
	HF	0.038	53	1

## Discussion

Our general findings from the behavior assays indicated that Prairie Rattlesnakes (*Crotalus viridis*), Mojave Rattlesnakes (*C. scutulatus*), and their hybrids (*C. scutulatus* × *viridis*) showed broadly similar behavior to each other, except in a few key traits. Prairie Rattlesnakes were more likely to rattle defensively compared to Mojave Rattlesnakes. Furthermore, hybrid individuals with a larger proportion of their genome derived from *C. viridis* also were more likely to rattle, indicating a potential genetic basis for this eponymous defensive trait. Across all three groups, snakes were generally hesitant to strike at threats and exhibited similar levels of exploration within a novel environment. Although *C. viridis* displayed the most correlated trait pairs, all three groups showed evidence of some syndromes between defensiveness and exploration/activity, although particulars of the syndromes differed across groups.

Because behavioral temperament can have a number of direct and indirect effects on fitness^47–49^, it is important to consider details of how these traits are expressed and measured in different contexts. The only behavioral trait we measured that differed significantly between groups was rattling behavior during the handling assay, an expression of defensiveness^50^. Defensive rattling, like most behaviors, is undoubtedly shaped by individual experience, ecological context, and genetic variability. The *C. viridis* we used in this study may have had more exposure to predators, as previous work found a higher number of predator encounters in the habitat occupied by *C. viridis* compared to areas occupied by *C. scutulatus*and hybrid individuals^51^. Thus, an increased propensity to rattle may be, in part, a product of stronger selection against individuals that do not react defensively toward potential predators or enemies^36,52–54^. However, an increase in the propensity to rattle was also significantly related to the proportion of the genome derived from *C. viridis* (HI) within hybrid individuals. Additionally, hybrid individuals with more prairie-like genomes were not spatially clustered, so it is unclear what environmental pressures would lead to this increased propensity to rattle.

Antipredator behaviors in a number of taxa have been shown to be heritable, with examples reported in insects^55,56^, mammals^57,58^, fish^59,60^, cephalopods^33^, birds^61,62^, and lizards^63^, but no studies exist for other non-avian reptiles. To our knowledge, the correlation we found between HI and rattling is the first empirical evidence pointing toward the potential for a genetic basis for the expression of an antipredator display in rattlesnakes. Future analyses incorporating genome-wide association studies and transcriptomic approaches could potentially identify genes or regions of the genome underpinning this unique aposematic behavior.

A large body of literature has shown that behavioral temperaments are often correlated across contexts^5^, and these syndromes can impact fitness either synergistically or antagonistically depending on context. Common syndromes seen across taxa include a positive correlation between boldness and general activity^7,24,25,64,65^, boldness and explorativeness^24,25,64,66–69^, and boldness and foraging^34,53,70^. Perhaps because it is associated with locating resources in the environment, the propensity to explore novel environments is often correlated with a number of other traits besides boldness, including general activity^64,71,72^, aggressiveness^68^, sociability^73^, and docility^74,75^. Our study adds to this body of work by showing evidence for a syndrome between the exploratory behaviors of rattlesnakes and how often they move within the landscape.

Since hybrid individuals are a product of two distinct parental genotypes, we hypothesized that syndromes would be generally weaker in hybrids^16,17^, but our results did not necessarily show that pattern. Although evidence for syndromes between defensiveness and exploration/activity was found in all groups, the particular behaviors that correlated across these contexts varied among the groups, and were not consistent among contexts within groups. *Crotalus viridis*, individuals that struck defensively exhibited higher levels of activity in novel environments by some measures (i.e., defensive strikers spent less time hiding under shelter or remaining motionless outside shelter), they also made fewer quadrat transitions. Hybrids (*C. scutulatus* × *viridis*) did not exhibit syndromes involving defensive striking, but individuals that rattled more spent less time motionless outside the shelter in the open field test, but also made fewer quadrat transitions. *Crotalus scutulatus*, individuals with a higher propensity to strike during the threat assay spent more time in the hidebox in the open field test, and also made fewer quadrant transitions.

The behaviors we measured in captivity, although commonly used across a number of taxa, can be challenging to interpret in an ecological context. The time an individual spends in a hidebox or the time it spends motionless could indicate general activity level since spending more time in a hidebox is also time spent inactive. Similarly, spending more time in a hidebox could also reflect an unwillingness to perform a risky behavior (exposing oneself in an unfamiliar environment), or shyness. In our assay, individuals that spent more time motionless or in hideboxes were less likely to strike when threatened directly, so perhaps these measures are more indicative of general activity. However, this interpretation is complicated by the fact that it was necessary to handle snakes immediately prior to the exploration assays, perhaps leaving them in a stressed state. Future studies using alternative methods would be necessary to assess the relationship more closely between time spent motionless and bold, explorative, and active temperaments. Such studies may be able to resolve some apparent contradictions in our results between syndromes involving “explorativeness” and “defensiveness”. For example, in *C. viridis* striking (defensiveness) was negatively correlated with time spent in hidebox (low explorativeness), time spent motionless (low explorativeness), and the number of quadrant transitions (high explorativeness). In this case, it is possible that *C. viridis* that are more likely to strike defensively are more “anxious” individuals, and that personality trait is manifesting differently in different contexts. In the open field box test, individuals that were active but not crossing quadrants were often climbing at the walls of arena—perhaps analogous to escape or pacing behavior. Less anxious snakes would either move around to different hideboxes (quadrant crossing and hiding), or sit in place out in the open (time spent motionless). Untangling these intricacies would likely require repeated testing of individuals across several different behavioral contexts, most feasibly using a captive colony of snakes.

Contrary to several previous studies in other vertebrate taxa, we found that hybridization of these lineages did not lead to the breakdown of behavioral syndromes; rather, hybrids expressed syndromes within the same contexts as parental lineages (i.e., defensiveness, explorativeness), but involving different combinations of traits than either parental type. Although hybridization can generate novel or extreme traits^18,20,76^, it may be difficult to predict a priori how recombination and introgression would affect the trait combinations related to temperament and syndromes. More research is needed in order to determine if general patterns apply across taxonomic groups.

In addition to differences between parental and hybrid snakes, we also found that temperament and syndromes varied across age groups. Although these shifts in temperament across age classes have been found in other reptiles, they have not previously been confirmed in rattlesnakes^77^. Across all groups juveniles did not rattle as readily as adult snakes. These patterns could be driven by higher predation pressure on juveniles, given that their smaller size results in less effective antipredator behaviors (e.g., shorter effective strike range, smaller venom glands) and a higher likelihood of being killed by a wider variety of predators^78^. Following this logic, adult snakes could be more likely to rattle and draw attention to themselves because of their enhanced ability to effectively strike and envenomate potential predators.

We also found that juvenile snakes did not exhibit the same behavioral syndrome between defensiveness and explorativeness that was found in adults. This was not entirely unexpected and has been reported in other vertebrate taxa. Even in cases where temperaments of juveniles are quantifiable, considerable shifts in behavior occur with ontogeny^10^. Additionally, selection could also be eliminating maladaptive behavioral combinations and thus leading to convergence of traits into behavioral syndromes as animals age^79^.

While we found several behavioral measures were correlated across contexts in the laboratory assay, there was much more limited evidence for behavioral syndromes that spanned the laboratory assays and field-based behaviors (although our sample size for these comparisons was much more limited). For all adult snakes combined, we found a significant relationship between increased exploration in the laboratory assay (number of quadrant transitions) and the field (more frequent movements). This syndrome indicates that the temperament of an individual could affect metrics associated with spatial ecology, as has been found in other systems^80^, and be used by researchers to make broad (but tentative) generalizations about the spatial ecology of individuals that have been assayed under laboratory conditions.

Although this relationship makes intuitive sense, supporting evidence in our study is still limited. Most of the movement and exploratory behaviors we measured in the laboratory were not correlated with any of the behaviors measured when those snakes were free-ranging. This may be in part because the laboratory assays and field measures of behavior represent very different contexts. Compared to an animal’s home range in the field, the standardized arena in which animals are tested in an exploratory assay is small and devoid of sensory cues that would be typical of a natural setting. The high degree of variability in spatial behaviors expressed by free-ranging snakes is likely associated with the greater degree of biotic and abiotic variability across natural systems, and might make it difficult to detect consistent relationships across contexts.

It is also possible that behavioral temperaments and syndromes are species- or population-specific. The suite of behaviors we measured in the laboratory assays was based off of a study of a captive colony of Western Rattlesnakes (*C. oreganus*), which displayed individual repeatability for these behaviors^40^. Even though Western, Prairie, and Mojave Rattlesnakes are closely related and diverged recently (Prairie/Western: 5.24 MYA; Prairie/Mojave: 5.47 MYA^43^), it is possible that interspecific variation exists in the degree and type of behavioral temperaments and syndromes, and that repeatability of behaviors or existence of syndromes found in other species and populations are not present at our site. Thus, although our relatively short-term study was able to document a suite of behaviors correlated across contexts, future work should validate how robust these behavioral syndromes are over time in an effort to further understand their relevance to individual fitness and population persistence.

Although we did not find that hybrids had generally weaker behavioral syndromes than parentals, we did find that hybrids (but not parentals) that moved more in the open field arena were also significantly less likely to rattle during the handling test, a syndrome that could indicate a lack of risk aversion in some individuals. Because predation risk for snakes appears to be highest when they are moving between sites^51^, individuals that move more and are less likely to exhibit appropriate antipredator responses may be more at risk from predation. Of the 18 *C. scutulatus* × *viridis *that were implanted with transmitters, seven (38.9%) of them died before the end of the tracking season. Although we could only directly confirm one of these was killed by a predator, predation is a likely source of mortality for the other individuals^45^. Qualitatively, this was a higher level of mortality than we observed for *C. scutulatus* (one out of 21, or 4.8% mortality) and *C. viridis *(two out of 17, or 11.8%)^45^. Hybrid individuals that tend to rattle less but moved more could be subject to higher levels of predation due to increased numbers of encounters and lower probability of engaging in effective anti-predator displays. However, more directed behavioral experiments paired with a long-term mark-recapture program would be necessary to quantitatively test the hypothesis that *C. scutulatus* × *viridis* are more susceptible to predation.

In conclusion, although the rattlesnakes we studied in this hybrid zone were broadly similar in temperament and syndromes, we did identify a few key differences that could potentially play a role in hybridization dynamics. Unexpectedly, we did not find significantly more variability in temperament within the hybrid lineage when compared to parentals. Nevertheless, this study is one of a few that integrates laboratory-based behavioral assays with behaviors expressed under natural conditions. This integration is extremely important for developing a holistic view of the utility of using data collected in captivity to understand natural populations. Future research could build on these findings by developing focused approaches to understand the role of temperament and syndromes in shaping survival and fitness, or reproductive isolation between lineages. Such efforts could yield important findings on the subtleties of how closely related species differ from each other, and how these differences affect reproductive isolation and the potential for speciation.

## Methods

### Study sites

The hybrid zone is located within the Cochise Filter Barrier (CFB)^[Figure 1 in 46^, a transitional region between the Chihuahuan and Sonoran deserts in the southwestern USA that is frequently implicated in lineage divergence associated with climatic and vegetation community shifts induced by glacial cycling^81^. Because there is not a major physical barrier separating the two deserts, the CFB is a region of frequent gene flow and hybridization between genetic groups of organisms^82,83^. The hybrid zone between *Crotalus scutulatus* and *C. viridis *occupies a valley between the Peloncillo and Animas Mountains in the extreme southwest of New Mexico, USA. Hybrid snakes are found in a narrow band (~ 12 km) of mosaic habitat in the center of the valley, an area defined by the transition from creosote lowland desert to the southwest to short-grass prairie to the northeast. Parental populations were located on either side of the bordering mountain ranges^84^. For further details on the hybrid zone, refer to^46^.

### Snake sampling and surgical procedures

We collected and sampled all rattlesnakes encountered via road and visual encounter surveys within and adjacent to the hybrid zone from 2019–2021^[Figure 1 in 45^. Capture, processing, and field telemetry/videography methods followed previous work^45,46^. In summary individuals were captured in the field, transported to the field station, sexed via hemipene probing, measured for a suite of morphometric characters, categorized as adult or juvenile (based off body size at initial capture and later confirmed using the distribution of Scaled Mass Index detailed in^46^; Figure S.3), tested for their laboratory behaviors, and either released at their initial point of capture or held for surgical implantation of radio transmitters and then released at their initial point of capture. All procedures were approved by the San Diego State University Institutional Animal Care and Use Committee (APF# 22-07-008 C), and animals were collected via a New Mexico Department of Game and Fish Scientific Collection permit (authorization number 3605). All procedures were also performed in accordance with ARRIVE guidelines: to reduce the number of animals involved in the experiment, animals were used as their own control; outcome measures are clearly defined; statistical analysis is fully detailed and the code is available on the following Dryad repository: 10.5061/dryad.5tb2rbpbd; the species, sex and age of animals are reported; and the experimental procedures are described in detail. All the methods were carried out in accordance with relevant guidelines and regulations.

We tested every subject for defensive and exploratory behaviors within 48 h of their initial capture. All testing was at 22–26 °C to minimize variation in behavior owing to body temperature. This temperature range is well within the range of naturally occurring temperatures at the hybrid zone, and overlaps with the field-active body temperature of actively hunting rattlesnakes^85^. Individuals were given at least four hours to acclimate to the room’s temperature prior to conducting tests. We followed the procedures in Gibert et al.^40^for conducting and scoring handling, exploratory, and threat assays. Snakes were assayed in a 1.2 × 1.2 × 1.2 m four-walled arena constructed from polyvinyl sheeting and dimly lit with indirect light. We divided the floor of the arena into four equal quadrants using black tape and placed a 10 × 5 × 3 cm black hidebox within each quadrant so that they were equidistance from each other and the center of the arena^[Figure 1 in 40^). After testing each snake, we cleaned the entire arena using a commercial cleaner (Lysol^®^) and the hideboxes with soap and water. We recorded each of the three assays (handling, exploratory, and threat) with a Sony Handycam^®^ (model DCR-SR80) mounted above the arena. When snakes were not being tested they were housed in their own cage, provided a hide box, and were given water *ad-libitum*.

### Handling assay

To assay defensive rattling, we simulated a predator attack by removing snakes with 1 m long metal tongs (Midwest Tongs^®^) from their temporary holding container after the 4-hour acclimation period and held them 1 m above the center of the arena at midbody for 30 s. At the 15 s mark we gently shook the snake back and forth twice. Once 30 s had elapsed, we released the snake in the center of the arena and left the area, starting the exploratory assay. Whether or not the snake rattled defensively during the handling assay was recorded from the video footage.

### Exploratory assay

Snakes were left to explore the arena undisturbed for 60 min. We then reviewed the video footage after each assay to quantify the duration of time the snake spent in a hidebox, the number of transitions between quadrants, and the duration it spent motionless outside of a hidebox. We considered the snake to have entered a hidebox or a new quadrant when the entire front half of the individual was either obscured from view within the hidebox or had broken the plane created by the black tape. We considered the snake to be motionless when no detectable movement of its head, body, or tail could be seen or heard.

### Threat assay

After the conclusion of the exploratory assay, we turned on an overhead light to fully illuminate the arena and, using metal tongs, removed all hideboxes and moved the snake into one corner of the arena. We then “threatened” the snake with an inflated balloon, around 0.25 m in diameter, on the end of another pair of metal tongs to create a visual, looming stimulus. For each assay the balloon tong was raised about 0.25 m into the air and tapped in the center of the floor of the arena five times, around 80 cm from the center of the snake with each tap taking around 1 s. We then slid the balloon along the floor of the arena and towards the head of the snake until it was around 40 cm from the snake. We then tapped the balloon tong on the floor of the arena another five times. After the initial tapping, we slid the balloon along the floor of the arena again until it touched the snout of the snake. If the snake did not strike at this point of the assay, we simulated a more immediate threat by tapping the balloon on the head of the snake five times. If at any point the snake struck at the balloon (even if the snake missed) the assay would end, and a positive strike response would be reported for that snake. Otherwise, the assay would end on the fifth tap of the balloon on the snake’s head and a negative response would be recorded, usually taking around 20 s. The threat assay was only performed on snakes captured during the 2020 and 2021 active seasons.

### Genetic assignments of individuals to parental species or hybrids

To assign snakes as parental or hybrid individuals we analyzed reduced-representation genomic data obtained from double-digest RAD sequencing (ddRADseq) and whole-genome sequencing. All sequenced samples were mapped to the *C. viridis *reference genome^43^. For individuals that had their whole genome sequenced, genomic data were downsampled to only include loci that overlapped with ddRADseq loci. Hybrid index (HI—proportion of the genome derived from *C. viridis*) scores for individuals were inferred across individuals using ADMIXTURE with K = 2. We classified any snake with an HI between 5 and 95% as a hybrid (see Fig. 2 in^46^ or Figure S4). Due to extraction or sequencing failures, we were not able to obtain HI estimates for six of the 185 snakes analyzed for their temperament and syndromes (based on face mask and tail banding patterns, and geography, one putative *C. scutulatus*, three putative *C. viridis*, and two putative *C. scutulatus* × *viridis*). We re-ran all analyses with and without these individuals and found no difference in the overall patterns (see Tables S1–S3 for analyses without these individuals), and thus chose to report the results that included the individuals with assignments based solely on morphology (Figure S5) and geographic locale.

### Statistical analyses

Behavioral assays were scored independently by two different observers blind to snake identity to minimize observer bias. If scores were similar (values within 15%), we averaged the scores of the two observers, but if there was a greater than 15% difference between observer scores, the video was scored by a third observer to achieve a consensus score (average of the two scores with < 15% difference).

To assess temperament and potential syndromes, we limited analyses to the behaviors that were found to be repeatable in previous research assessing repeatability of temperament traits in *C. oreganus*^40^. The traits identified by Gibert et al.^40^are also broadly similar to other temperament traits that have been found to be individually repeatable across a broad sample of snakes, lizards, and other vertebrates^86^ (including the conspecific *C. atrox*^41^). Thus, we scored rattling behavior (whether snake rattled or not during the handling assay) and defensive strike behavior (whether snake struck or not during the threat assay) as traits indicative of defensive/passive temperaments^77,87–91^. During the exploratory test, we scored the proportion of time a snake spent in a hidebox, the proportion of time spent motionless (not moving, but not in hidebox), and the number of times the snake transitioned to a new quadrant. Behaviors like these displayed in open-field tests are typically considered indicative of explorative/non-explorative temperaments^4,92^.

We created a suite of models using a Generalized Linear Model (GLM) framework to assess variation in defensive and exploratory temperaments across genetic groups (*C. scutulatus*, *C. viridis*, and *C. scutulatus* × *viridis*). Four models were constructed for each of the response variables (defensive rattling, proportion of time spent in a hidebox, number of quadrant transitions, proportion of time spent motionless, and striking), with each modeling the predictor variables of genetic group, group + age (adult or juvenile), group * age, and group * age + sex. We then used Akaike information criterion (AIC_C_) to select which of the four models best fit the data. If more than one model was within two ΔAIC_C_of the best model, then we used the simplest model for analysis (the model with fewest predictor variables). We used a binomial distribution for modelling rattling, striking, and proportion of time motionless, a beta distribution for proportion of time in hidebox, and a negative binomial distribution for the number of quadrant transitions (overdispersed count data). Time spent in hidebox was zero-inflated, so we performed the transformation recommended by Smithson and Verkuilen^93^. We used Tukey tests for *post-hoc* comparisons and Levene’s test to compare variation between groups in exploratory behaviors. To assess if there was a relationship between the value of the hybrid index and temperament, we repeated the analyses using only hybrid snakes and incorporating HI as a fixed factor.

Due to non-normality of predictor variables and the binary nature of response variables, we were not able to use a correlation framework for analyzing behavioral syndromes between defensiveness and explorativeness, and instead used GLMs to test for significant relationships between behavioral traits within each group^15,40,73,94^. For each group, we constructed binomial GLMs with the three exploratory behaviors as the fixed factors and either rattling or defensive striking as the response variable. Lastly, if any previous models indicated age was a significant factor, we constructed independent models for adults and juveniles.

We also assessed potential syndromes between behaviors measured in laboratory assays and movement and hunting behaviors measured while individuals were free-ranging. These analyses were constrained in sample size, as we could only include the subset of radio-tagged individuals with enough spatial and hunting data for statistical analyses. These snakes were captured, implanted with radio-transmitters, released back at their point of capture and tracked for 1–2 active seasons. Each time the snake was found a GPS point was taken for spatial ecology/movement analysis. If the snake was found hunting, then a fixed field-videography set-up was deployed to document the entire hunting event for hunting behavior and diet analysis^45^. For this analysis we assessed relationships between explorative behaviors measured in captive assays and a suite of behaviors related to space use and foraging that we quantified in previous studies^45,46^, including: average distance moved per day (DMD), average number of days between movements (FM), patchiness of space use (number of 50% isopleths identified by Brownian Bridge Kernel Density Estimators (bbKDE), rate of chemosensory probing (NPR), prey encounter frequency (PE), time of day of hunting site abandonment (AT), and the frequency of nights that the snakes were found hunting (HF). We performed a Spearman Correlation test on the spatial and hunting behaviors and each of the three exploratory behaviors for each group and (in order to maximize sample size) for all groups combined. We adjusted p-values with a Holm’s adjustment to account for multiple comparisons. Following others^65,70,95^, we considered a syndrome to exist between two behaviors if the correlation between them was significant and if |r| > 0.3.

All behavioral scoring was done from the video footage *post-hoc *using BORIS v. 7.4.11^96^. All statistical analyses were done in R v. 3.6.3^97^using the packages tidyverse^98^, Hmisc^99^, nortest^100^, psych^101^, betareg^102^, ggplot2^103^, emmeans^104^, MuMIn^105^, performance^106^.

## Electronic supplementary material

Below is the link to the electronic supplementary material.

Supplementary Material 1

## Data Availability

Raw sequence reads for RADseq data are available under the NCBI SRA (sequence read archive) BioProjectID number: PRJNA1010815. The final VCF alignment file used for hybrid index analysis; individual behavior scores are available on Dryad: 10.5061/dryad.5tb2rbpbd.

## References

[1] MacKinlay, R. D. & Shaw, R. C. A systematic review of animal personality in conservation science. *Conserv. Biol.* **e13935**10.1111/cobi.13935 (2022). 10.1111/cobi.13935 PMC10084254 35561041

[2] Bell, A. M., Hankison, S. J. & Laskowski, K. L. The repeatability of behaviour: a meta-analysis. *Anim. Behav.* **77**, 771–783 (2009). 24707058 10.1016/j.anbehav.2008.12.022 PMC3972767

[3] van Oers, K., de Jong, G., van Noordwijk, A. J., Kempenaers, B. & Drent, P. J. Contribution of genetics to the study of animal personalities: a review of case studies. *Behaviour* **142**, 1185–1206 (2005).

[4] Carter, A. J., Feeney, W. E., Marshall, H. H., Cowlishaw, G. & Heinsohn, R. Animal personality: what are behavioural ecologists measuring? *Biol. Rev.* **88**, 465–475 (2013). 23253069 10.1111/brv.12007

[5] Sih, A., Bell, A. M., Johnson, J. C. & Ziemba, R. E. Behavioral syndromes: an integrative overview. *Q. Rev. Biol.* **79**, 241–277 (2004). 15529965 10.1086/422893

[6] Dingemanse, N. J., Kazem, A. J. N., Réale, D. & Wright, J. Behavioural reaction norms: animal personality Meets individual plasticity. *Trends Ecol. Evol.* **25**, 81–89 (2010). 19748700 10.1016/j.tree.2009.07.013

[7] Biro, P. A. & Stamps, J. A. Are animal personality traits linked to life-history productivity? *Trends Ecol. Evol.* **23**, 361–368 (2008). 18501468 10.1016/j.tree.2008.04.003

[8] Schuett, W., Tregenza, T. & Dall, S. R. X. Sexual selection and animal personality. *Biol. Rev.* **85**, 217–246 (2010). 19922534 10.1111/j.1469-185X.2009.00101.x

[9] Sih, A. et al. Animal personality and state-behaviour feedbacks: a review and guide for empiricists. *Trends Ecol. Evol.* **30**, 50–60 (2015). 25498413 10.1016/j.tree.2014.11.004

[10] Cabrera, D., Nilsson, J. R. & Griffen, B. D. The development of animal personality across ontogeny: a cross-species review. *Anim. Behav.* **173**, 137–144 (2021).

[11] Laskowski, K. L., Chang, C. C. & Sheehy, K. Aguin Tild Aga, J. Consistent individual behavioral variation: what do we know and where are we going? *Annu. Rev. Ecol. Evol. Syst.* **53**, 161–182 (2022).

[12] Rieseberg, L. H. et al. Hybridization and the colonization of novel habitats by annual sunflowers. *Genetica* **129**, 149–165 (2007). 16955330 10.1007/s10709-006-9011-y PMC2442915

[13] Simon, A., Bierne, N. & Welch, J. J. Coadapted genomes and selection on hybrids: Fisher’s geometric model explains a variety of empirical patterns. *Evol. Lett.* **2**, 472–498 (2018). 30283696 10.1002/evl3.66 PMC6145440

[14] Horta-Lacueva, Q. J. B., Benhaïm, D., Morrissey, M. B., Snorrason, S. S. & Kapralova, K. H. Animal personality adds complexity to the processes of divergence between sympatric morphs of Arctic Charr. *Anim. Behav.* **175**, 57–73 (2021).

[15] Johnson, J. B., Culumber, Z. W., Easterling, R. & Rosenthal, G. G. Boldness and predator evasion in naturally hybridizing swordtails (Teleostei: *Xiphophorus*). *Curr. Zool.* **61**, 596–603 (2015).

[16] Islam, S. S., Wringe, B. F., Bradbury, I. R. & Fleming, I. A. Behavioural variation among divergent European and North American farmed and wild Atlantic salmon (*Salmo salar*) populations. *Appl. Anim. Behav. Sci.* **230**, 105029 (2020).

[17] Hosoya, S., Suetake, H., Suzuki, Y. & Kikuchi, K. Genetic basis underlying behavioral correlation between Fugu *Takifugu rubripes* and a closely related species, *Takifugu niphobles*. *Behav. Genet.* **45**, 560–572 (2015). 26067468 10.1007/s10519-015-9728-4

[18] Rieseberg, L. H., Archer, M. A. & Wayne, R. K. Transgressive segregation, adaptation and speciation. *Heredity (Edinb)*. **83**, 363–372 (1999). 10583537 10.1038/sj.hdy.6886170

[19] Stelkens, R. & Seehausen, O. Genetic distance between species predicts novel trait expression in their hybrids. *Evol. (N Y)*. **63**, 884–897 (2009). 10.1111/j.1558-5646.2008.00599.x 19220450

[20] Harrison, R. G. & Larson, E. L. Hybridization, introgression, and the nature of species boundaries. *J. Hered*. **105**, 795–809 (2014). 25149255 10.1093/jhered/esu033

[21] Seehausen, O. Hybridization and adaptive radiation. *Trends Ecol. Evol.* **19**, 198–207 (2004). 16701254 10.1016/j.tree.2004.01.003

[22] Ingley, S. J. & Johnson, J. B. Animal personality as a driver of reproductive isolation. *Trends Ecol. Evol.* **29**, 369–371 (2014). 24837793 10.1016/j.tree.2014.04.008

[23] Harcourt, J. L., Sweetman, G., Johnstone, R. A. & Manica, A. Personality counts: the effect of boldness on shoal choice in Three-spined sticklebacks. *Anim. Behav.* **77**, 1501–1505 (2009).

[24] Michelangeli, M., Wong, B. B. M. & Chapple, D. G. It’s a trap: sampling bias due to animal personality is not always inevitable. *Behav. Ecol.* **27**, 62–67 (2016).

[25] Wilson, A. D. M. & Godin, J. G. J. Boldness and behavioral syndromes in the Bluegill sunfish, *Lepomis macrochirus*. *Behav. Ecol.* **20**, 231–237 (2009).

[26] Sinn, D. L. & Moltschaniwskyj, N. A. Personality traits in dumpling squid (*Euprymna tasmanica*): context-specific traits and their correlation with biological characteristics. *J. Comp. Psychol.* **119**, 99–110 (2005). 15740434 10.1037/0735-7036.119.1.99

[27] Reaney, L. T. & Backwell, P. R. Y. Risk-taking behavior predicts aggression and mating success in a fiddler crab. *Behav. Ecol.* **18**, 521–525 (2007).

[28] Breck, S. W., Poessel, S. A., Mahoney, P. & Young, J. K. The intrepid urban Coyote: a comparison of bold and exploratory behavior in Coyotes from urban and rural environments. *Sci. Rep.* **9**, 2104 (2019). 30765777 10.1038/s41598-019-38543-5 PMC6376053

[29] del Mar Delgado, M. & Penteriani, V. Behavioral States help translate dispersal movements into Spatial distribution patterns of floaters. *Am. Nat.* **172**, 475–485 (2008). 18729727 10.1086/590964

[30] Fraser, D. F., Gilliam, J. F., Daley, M. J., Le, A. N. & Skalski, G. T. Explaining leptokurtic movement distributions: intrapopulation variation in boldness and exploration. *Am. Nat.* **158**, 124–135 (2001). 18707341 10.1086/321307

[31] Toscano, B. J., Gownaris, N. J., Heerhartz, S. M. & Monaco, C. J. Personality, foraging behavior and specialization: integrating behavioral and food web ecology at the individual level. *Oecologia* **182**, 55–69 (2016). 27170290 10.1007/s00442-016-3648-8

[32] Stuber, E. F., Carlson, B. S. & Jesmer, B. R. Spatial personalities: a meta-analysis of consistent individual differences in Spatial behavior. *Behav. Ecol.* **33**, 477–486 (2022).

[33] Sinn, D. L., Apiolaza, L. A. & Moltschaniwskyj, N. A. Heritability and fitness-related consequences of squid personality traits. *J. Evol. Biol.* **19**, 1437–1447 (2006). 16910975 10.1111/j.1420-9101.2006.01136.x

[34] Wilson, A. D. M. & Stevens, E. D. Consistency in context-specific measures of shyness and boldness in rainbow trout, *Oncorhynchus mykiss*. *Ethology* **111**, 849–862 (2005).

[35] Quinn, J. L. & Cresswell, W. Personality, anti-predation behaviour and behavioural plasticity in the chaffinch *Fringilla coelebs*. *Behaviour* **142**, 1377–1402 (2005).

[36] Réale, D. & Festa-Bianchet, M. Predator-induced natural selection on temperament in Bighorn Ewes. *Anim. Behav.* **65**, 463–470 (2003).

[37] Stapley, J. & Keogh, J. S. Behavioral syndromes influence mating systems: floater pairs of a Lizard have heavier offspring. *Behav. Ecol.* **16**, 514–520 (2005).

[38] Sinn, D. L., Gosling, S. D. & Moltschaniwskyj, N. A. Development of Shy/bold behaviour in Squid: context-specific phenotypes associated with developmental plasticity. *Anim. Behav.* **75**, 433–442 (2008).

[39] Collins, S. M. et al. Bibliometric investigation of the integration of animal personality in conservation contexts. *Conserv. Biol.* e14021. 10.1111/cobi.COBI14021 (2022). 10.1111/cobi.14021 36285603

[40] Gibert, R. G., Maag, D. W., Sanders, L. N. & Clark, R. W. Investigating personality in vipers: individual rattlesnakes exhibit consistent behavioral responses in defensive and exploratory contexts. *Behav. Ecol. Sociobiol.* **76**, 132 (2022).

[41] Da Cunha, O., Horne, L. M. & Johnson, J. D. Personally rattled: a unique protocol to support the presence of personality and behavioral syndromes in rattlesnakes. *Behav. Ecol. Sociobiol.* **77**, 115 (2023).

[42] Schield, D. R. et al. Cryptic genetic diversity, population structure, and gene flow in the Mojave rattlesnake (*Crotalus scutulatus*). *Mol. Phylogenet Evol.* **127**, 669–681 (2018). 29902574 10.1016/j.ympev.2018.06.013

[43] Schield, D. R. et al. Allopatric divergence and secondary contact with gene flow: a recurring theme in rattlesnake speciation. *Biol. J. Linn. Soc.* **128**, 149–169 (2019).

[44] Nikolakis, Z. L. et al. Evidence that genomic incompatibilities and other multilocus processes impact hybrid fitness in a rattlesnake hybrid zone. *Evol. (N Y)*. **76**, 2513–2530 (2022). 10.1111/evo.14612 36111705

[45] Maag, D. W., Francioli, Y. Z., Castoe, T. A., Schuett, G. W. & Clark, R. W. The spatial ecology of Mojave Rattlesnakes (*Crotalus scutulatus*), Prairie Rattlesnakes (*C. viridis*), and their hybrids in southwestern New Mexico. *Biol. J. Linn. Soc.* blae037 (2024). 10.1002/ece3.10683 PMC10630157 38020675

[46] Maag, D. W. et al. Hunting behavior and feeding ecology of Mojave rattlesnakes (*Crotalus scutulatus*), prairie rattlesnakes (*C. viridis*), and their hybrids in Southwestern new Mexico. *Ecol. Evol.* **13**, e10683 (2023). 38020675 10.1002/ece3.10683 PMC10630157

[47] Ballew, N. G., Mittelbach, G. G. & Scribner, K. T. Fitness consequences of boldness in juvenile and adult largemouth bass. *Am. Nat.* **189**, 396–406 (2017). 28350493 10.1086/690909

[48] Smith, B. R. & Blumstein, D. T. Fitness consequences of personality: a meta-analysis. *Behav. Ecol.* **19**, 448–455 (2008).

[49] von Merten, S., Dingemanse, N. J., Mathias, M. L. & Rychlik, L. Individual behavior, behavioral stability, and Pace of life within and among five shrew species. *Behav. Ecol. Sociobiol.* **74**, 15 (2020).

[50] Klauber, L. M. The Rattle. in *Rattlesnakes: their habits, life histories, and influence on mankind* 249–322 (1956).

[51] Maag, D. & Clark, R. Safety in coils: predation rates of ambush hunting rattlesnakes are extremely low. *Amphib Reptil*. **43**, 425–430 (2022).

[52] Kashon, E. A. F. & Carlson, B. E. Consistently bolder turtles maintain higher body temperatures in the field but May experience greater predation risk. *Behav. Ecol. Sociobiol.* **72**, 9 (2018).

[53] Carter, A. J., Goldizen, A. W. & Tromp, S. A. Agamas exhibit behavioral syndromes: bolder males Bask and feed more but May suffer higher predation. *Behav. Ecol.* **21**, 655–661 (2010).

[54] Bell, A. M. & Sih, A. Exposure to predation generates personality in threespined sticklebacks (*Gasterosteus aculeatus*). *Ecol. Lett.* **10**, 828–834 (2007). 17663716 10.1111/j.1461-0248.2007.01081.x

[55] Guzmán-Novoa, E., Hunt, G. J., Uribe, J. L., Smith, C. & Arechavaleta-Velasco, M. E. Confirmation of QTL effects and evidence of genetic dominance of honeybee defensive behavior: results of colony and individual behavioral assays. *Behav. Genet.* **32**, 96–102 (2002). 10.1023/a:1015245605670 12036115

[56] Nakayama, S., Nishi, Y. & Miyatake, T. Genetic correlation between behavioural traits in relation to death-feigning behaviour. *Popul. Ecol.* **52**, 329–335 (2010).

[57] Tay, N. E., Warburton, N. M., Moseby, K. E. & Fleming, P. A. Predator escape behaviour in threatened marsupials. *Anim. Conserv.* 1–14. 10.1111/acv.12847 (2023).

[58] Gammie, S. C., Garland, T. & Stevenson, S. A. Artificial selection for increased maternal defense behavior in mice. *Behav. Genet.* **36**, 713–722 (2006). 16676225 10.1007/s10519-006-9071-x PMC2423941

[59] Kim, S. Y. & Velando, A. Phenotypic integration between antipredator behavior and camouflage pattern in juvenile sticklebacks. *Evol. (N Y)*. **69**, 830–838 (2015). 10.1111/evo.12600 25572122

[60] Satterfield, D. & Johnson, D. W. Local adaptation of antipredator behaviors in populations of a temperate reef fish. *Oecologia* **194**, 571–584 (2020). 32964291 10.1007/s00442-020-04757-y

[61] Jiang, Y. & Møller, A. P. Escape from predators and genetic variance in birds. *J. Evol. Biol.* **30**, 2059–2067 (2017). 28898481 10.1111/jeb.13175

[62] Bize, P., Diaz, C. & Lindström, J. Experimental evidence that adult antipredator behaviour is heritable and not influenced by behavioural copying in a wild bird. *Proc. R Soc. B Biol. Sci.* **279**, 1380–1388 (2012). 10.1098/rspb.2011.1789 PMC3282373 21976691

[63] Baxter-Gilbert, J., Riley, J. L. & Whiting, M. J. Runners and fighters: clutch effects and body size drive innate antipredator behaviour in hatchling lizards. *Behav. Ecol. Sociobiol.* **72**, 97 (2018).

[64] Michelangeli, M., Chapple, D. G., Goulet, C. T., Bertram, M. G. & Wong, B. M. Behavioral syndromes vary among geographically distinct populations in a reptile. *Behav. Ecol.* **30**, 393–401 (2019).

[65] Lukas, J. et al. Consistent behavioral syndrome across seasons in an invasive freshwater fish. *Front. Ecol. Evol.* **8**, 583670 (2021).

[66] Kudo, H., Nishizawa, H., Uchida, K. & Sato, K. Boldness–exploration behavioral syndrome in wild sub-adult green sea turtles caught at Oita, Japan. *Appl. Anim. Behav. Sci.* **236**, 105216 (2021).

[67] Xu, W. et al. Environmental complexity during early life shapes average behavior in adulthood. *Behav. Ecol.* **32**, 105–113 (2021).

[68] Schabacker, T. et al. In situ novel environment assay reveals acoustic exploration as a repeatable behavioral response in migratory bats. *Sci. Rep.* **11**, 8174 (2021). 33854128 10.1038/s41598-021-87588-y PMC8046999

[69] Majelantle, T. L. et al. Aggression, boldness, and exploration personality traits in the subterranean naked Mole-Rat (*Heterocephalus glaber*) disperser morphs. *Animals* **12**, 3083 (2022). 36428311 10.3390/ani12223083 PMC9686569

[70] Nyqvist, M. J., Gozlan, R. E., Cucherousset, J. & Britton, J. R. Behavioural syndrome in a solitary predator is independent of body size and growth rate. *PLoS One*. **7**, e31619 (2012). 22363687 10.1371/journal.pone.0031619 PMC3282768

[71] Muraco, J. J., Monroe, D. J., Aspbury, A. S. & Gabor, C. R. Do females in a unisexual-bisexual species complex differ in their behavioral syndromes and cortisol production? *Biology (Basel)*. **10**, 186 (2021). 33802259 10.3390/biology10030186 PMC8001229

[72] Ferderer, A., Davis, A. R. & Wong, M. Y. L. Temperature and body size influence personality and behavioural syndromes in an invasive crayfish. *Anim. Behav.* **190**, 187–198 (2022).

[73] Dhellemmes, F., Finger, J. S., Laskowski, K. L., Guttridge, T. L. & Krause, J. Comparing behavioural syndromes across time and ecological conditions in a free-ranging predator. *Anim. Behav.* **162**, 23–33 (2020).

[74] Agnani, P., Thomson, J., Schradin, C. & Careau, V. The fast and the curious II: performance, personality, and metabolism in Karoo Bush rats. *Behav. Ecol. Sociobiol.* **74**, 123 (2020).

[75] Underhill, V. et al. Personality and behavioral syndromes in two peromyscus species: presence, lack of state dependence, and lack of association with home range size. *Behav. Ecol. Sociobiol.* **75**, 9 (2021).

[76] Stelkens, R. B., Schmid, C., Selz, O. & Seehausen, O. Phenotypic novelty in experimental hybrids is predicted by the genetic distance between species of cichlid fish. *BMC Evol. Biol.* **9**, 283 (2009). 19961584 10.1186/1471-2148-9-283 PMC2796671

[77] Simkova, O., Frýdlová, P., Zampachová, B., Frynta, D. & Landová, E. Development of behavioural profile in the Northern common Boa (*Boa imperator*): repeatable independent traits or personality? *PLoS ONE* **12**, e0177911 (2017). 28542424 10.1371/journal.pone.0177911 PMC5443515

[78] Klauber, L. M. Enemies of rattlesnakes. in *Rattlesnakes: their Habits, Life Histories, and Influence on Mankind* 1064–1115 (University of California Press, Ltd., 1956).

[79] Adriaenssens, B. & Johnsson, J. I. Natural selection, plasticity and the emergence of a behavioural syndrome in the wild. *Ecol. Lett.* **16**, 47–55 (2013). 23034098 10.1111/ele.12011

[80] Spiegel, O., Leu, S. T., Bull, C. M. & Sih, A. What’s your move? Movement as a link between personality and Spatial dynamics in animal populations. *Ecol. Lett.* **20**, 3–18 (2017). 28000433 10.1111/ele.12708

[81] Van Devender, T. R., Betancourt, J. L. & Wimberly, M. Biogeographic implications of a packrat midden sequence from the Sacramento mountains, south-central new Mexico. *Quat Res.* **22**, 344–360 (1984).

[82] Pyron, R. A. & Burbrink, F. T. Hard and soft allopatry: physically and ecologically mediated modes of geographic speciation. *J. Biogeogr.* **37**, 2005–2015 (2010).

[83] Castoe, T. A., Spencer, C. L. & Parkinson, C. L. Phylogeographic structure and historical demography of the Western Diamondback rattlesnake (*Crotalus atrox*): A perspective on North American desert biogeography. *Mol. Phylogenet Evol.* **42**, 193–212 (2007). 16934495 10.1016/j.ympev.2006.07.002

[84] Zancolli, G. et al. Is hybridization a source of adaptive venom. *Toxins (Basel)*. **8**, 188 (2016). 27322321 10.3390/toxins8060188 PMC4926154

[85] Putman, B. J. & Clark, R. W. Behavioral thermal tolerances of free-ranging rattlesnakes (*Crotalus oreganus*) during the summer foraging season. *J. Therm. Biol.* **65**, 8–15 (2017). 28343580 10.1016/j.jtherbio.2017.01.012

[86] Waters, M. R., Bowers, B. B. & Burghardt, G. M. Personality and individuality in reptile behavior. In *Personality in Nonhuman Animals* (eds Vonk, J. et al.) 153–184 (Springer, 2017).

[87] Maillet, Z., Halliday, W. D. & Blouin-Demers, G. Exploratory and defensive behaviours change with sex and body size in Eastern garter snakes (*Thamnophis sirtalis*). *J. Ethol.* **33**, 47–54 (2015).

[88] Scudder, R. M. & Burghardt, G. M. A comparative study of defensive behavior in three sympatric species of water snakes (*Nerodia*). *Z. Tierpsychol*. **63**, 17–26 (1983).

[89] Goode, M. J. & Duvall, D. Body temperature and defensive behaviour of free-ranging prairie rattlesnakes, *Crotalus viridis viridis*. *Anim. Behav.* **38**, 360–362 (1988).

[90] Arnold, S. J. & Bennett, A. F. Behavioural variation in natural populations. III: antipredator displays in the garter snake *Thamnophis radix*. *Anim. Behav.* **32**, 1108–1118 (1984).

[91] Herzog, H. A., Bowers, B. B. & Burghardt, G. M. Stimulus control of antipredator behavior in newborn and juvenile garter snakes (*Thamnophis*). *J. Comp. Psychol.* **103**, 233–242 (1989).

[92] Perals, D., Griffin, A. S., Bartomeus, I. & Sol, D. Revisiting the open-field test: what does it really tell Us about animal personality? *Anim. Behav.* **123**, 69–79 (2017).

[93] Smithson, M. & Verkuilen, J. A better lemon squeezer? Maximum-likelihood regression with beta-distributed dependent variables. *Psychol. Methods*. **11**, 54–71 (2006). 16594767 10.1037/1082-989X.11.1.54

[94] De Meester, G., Pafilis, P. & Van Damme, R. Bold and Bright: shy and supple? The effect of habitat type on personality–cognition covariance in The Aegean wall Lizard (*Podarcis erhardii*). *Anim. Cogn.* **25**, 745–767 (2022). 35037121 10.1007/s10071-021-01587-0

[95] Pruitt, J. N. et al. Population differences in behaviour are explained by shared within-population trait correlations. *J. Evol. Biol.* **23**, 748–756 (2010). 20149021 10.1111/j.1420-9101.2010.01940.x

[96] Friard, O. & Gamba, M. BORIS: a free, versatile open-source event-logging software for video/audio coding and live observations. *Methods Ecol. Evol.* **7**, 1325–1330 (2016).

[97] R Core Team. R: A language and environment for statistical computing (2021).

[98] Wickham, H. et al. Welcome to the {tidyverse}. *J. Open. Source Softw.* **4**, 1686 (2019).

[99] Harrell, F. E. Jr *With Contributions from Charles Dupont & Many Others* (Harrell Miscellaneous, 2021).

[100] Gross, J. & Ligges, U. nortest: Tests for normality (2015).

[101] Revelle, W. psych: Procedures for Personality and Psychological Research (2021).

[102] Francisco, C. N. & Zeileis, A. Beta regression in R. *J. Stat. Softw.* **34**, 1–24 (2010).

[103] Wickham, H. ggplot2: elegant graphics for data analysis (2016).

[104] Lenth, R. V. Emmeans: estimated marginal means, aka least-squares means. 10.1080/00031305.1980.10483031>License (2021).

[105] Barton, K. & MuMIn Multi-Model Inference (2020).

[106] Ludeck, D., Ben-Shachar, M. S., Patil, I., Waggoner, P. & Makowski, D. performance: and R package for assessment, comparison and testing of statistical models. 10.21105/joss.03139 (2021).

