# Supplementary material for: Variation in defensive and exploratory behaviors across a rattlesnake (Crotalus scutulatus × viridis) hybrid zone in southwestern New Mexico
Source: Sci Rep. 2025 Apr 8;15:11989. doi: 10.1038/s41598-025-96155-8 (PMC11978939; doi:10.1038/s41598-025-96155-8)
Supplement: Supplementary file 1 — Supplementary Material 1 [file 41598_2025_96155_MOESM1_ESM.docx]

**Variation in defensive and exploratory behaviors across a rattlesnake (*Crotalus scutulatus* x *viridis*) hybrid zone in southwestern New Mexico**

Dylan W. Maag*^1, 2^, Yannick Z. Francioli^3^, Matthew T. H. Goetz^4^, Lea N. Sanders^4^, Xochitl Lopez^4^, Todd A. Castoe^3^, Gordon W. Schuett^5, 6^, and Rulon W. Clark^4, 6^

^1^Department of Evolution, Ecology, and Organismal Biology, University of California, Riverside, Riverside, CA

^2^Department of Life, Earth, and Environmental Sciences, West Texas A&M University, Canyon, TX

^3^Department of Biology, University of Texas at Arlington, Arlington, TX

^4^Department of Biology, San Diego State University, San Diego, CA

^5^Department of Biology | Neuroscience Institute, Georgia State University, Atlanta, GA

^6^Chiricahua Desert Museum, Rodeo, NM

Correspondence: address, WTAMU Natural Sciences Building 329, Canyon, TX 79016; email, [dr.dylan.maag@gmail.com](mailto:dr.dylan.maag@gmail.com) or [dmaag@wtamu.edu](mailto:dmaag@wtamu.edu)

**Supplementary Information**


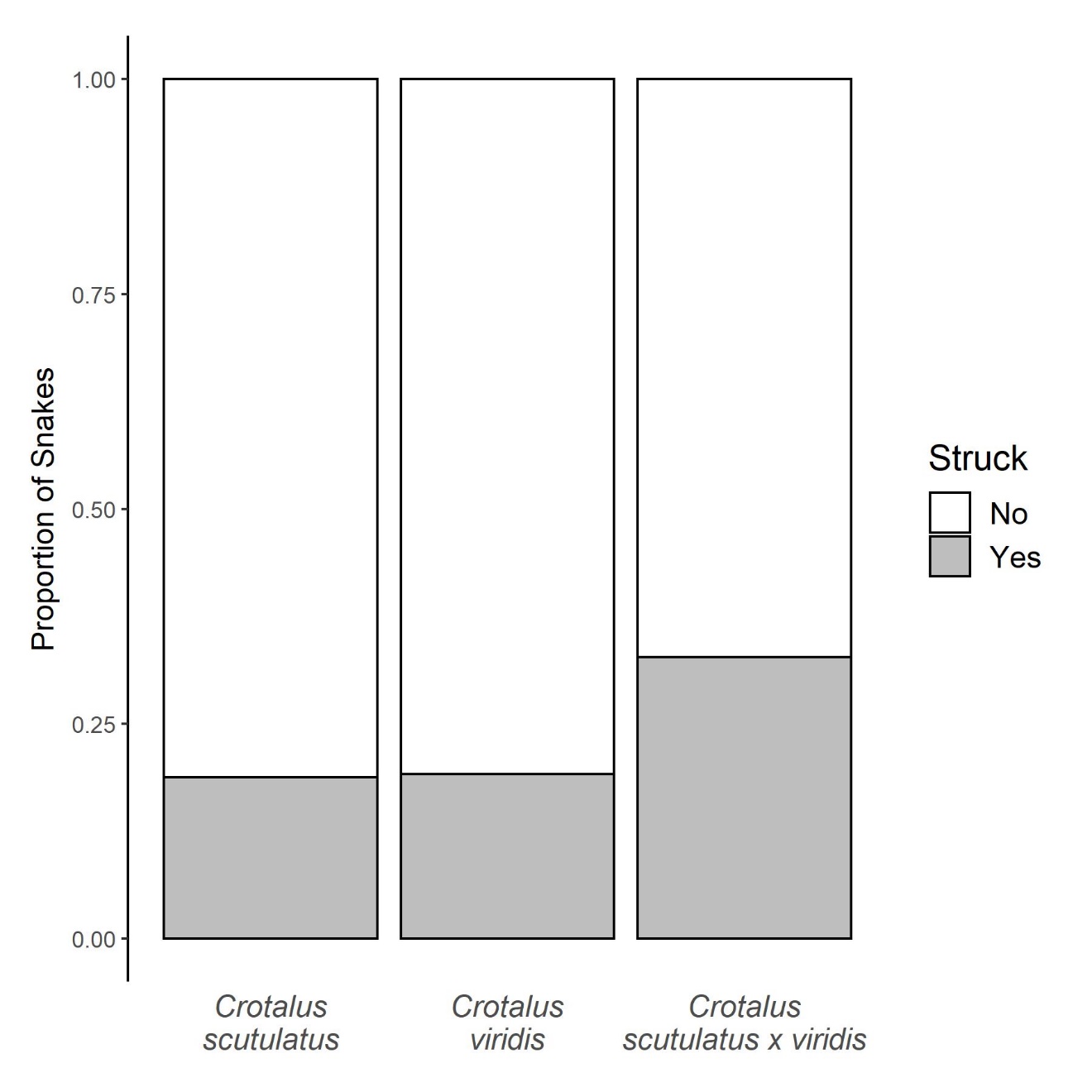


**Figure S.1:** Bar graph of the proportions of snakes that struck defensively during the threat assay. Only 24.6% of snakes struck during the assay and no differences were seen between the genetic groups (X^2^ = 3.26, df = 2, p = 0.196). Sample sizes: *Crotalus scutulatus* = 32, *C. viridis* = 47, *C.* *scutulatus* × *viridis* = 55.


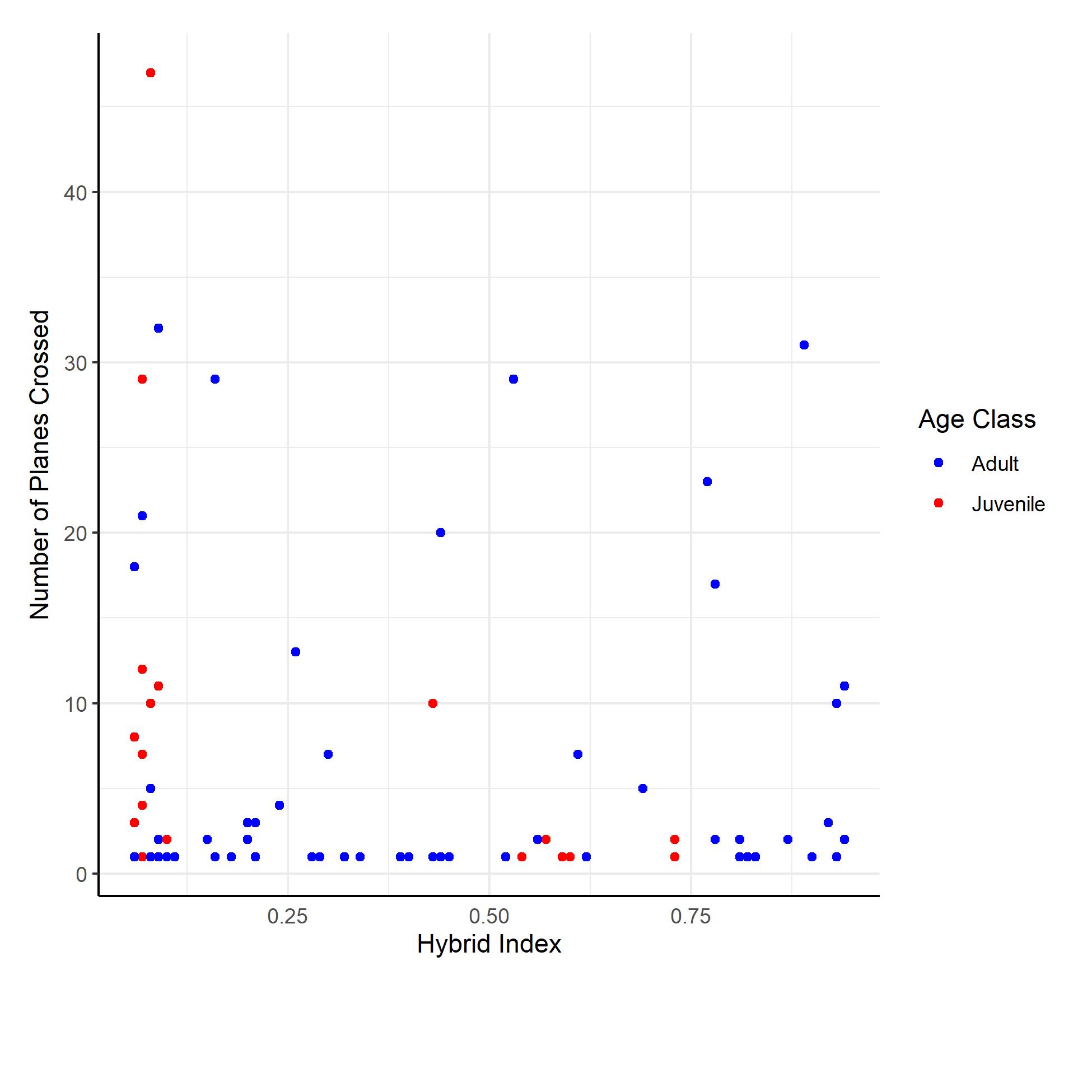


**Figure S.2:** Scatter plot showing the interaction between the age class of a hybrid snake and how many quadrant transitions it performed. This was a significant relationship (X^2^ = 7.782, df = 1, p = 0.005) but is likely driven by two juveniles that performed 47 and 29 transitions. Sample sizes: Adults = 56, Juveniles = 29. These two snakes were confirmed to be outliers by the Minimum Covariance Determinant method since the data was heavily skewed. After the removal of those two individuals the number of quadrant transitions was not significantly related to the Hybrid Index of the snakes (X^2^ = 0.138, df = 1, p = 0.710) or their age (X^2^ = 3.513, df = 1, p = 0.061). Additionally, there was no significant interaction between the Hybrid Index and age of the snakes (X^2^ = 3.232, df = 1, p = 0.072).


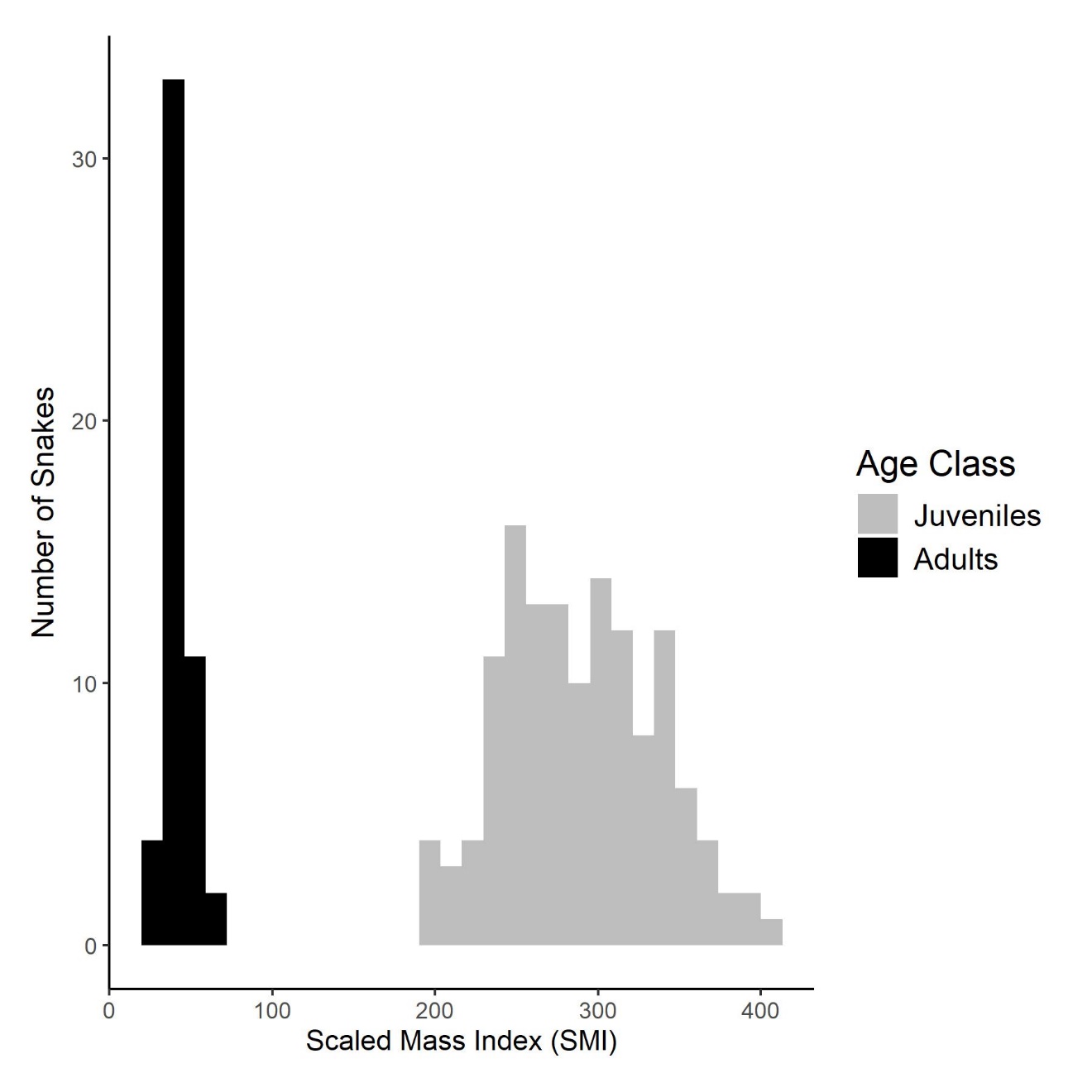


**Figure S.3:** Histogram showing the number of snakes in either the Adult or Juvenile, showing clear separation of age class based on their Scaled Mass Index calculated in^46^.


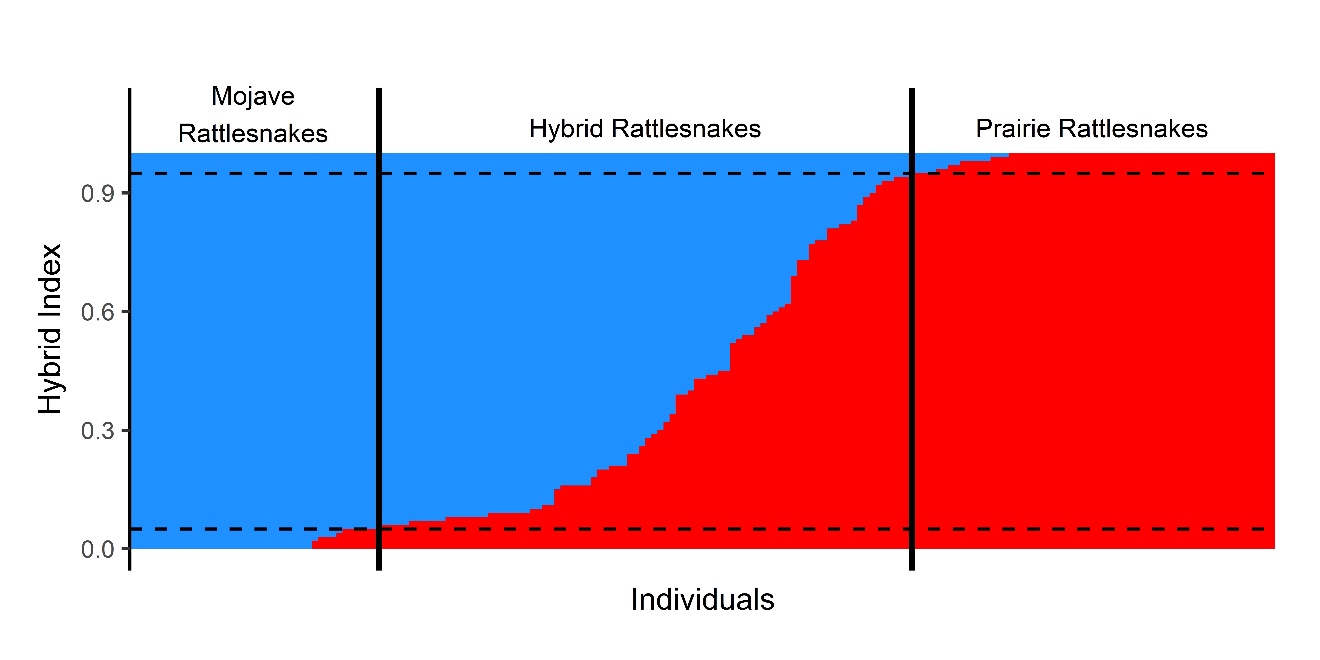


**Figure S.4:** This is Figure 2 in ^46^. Hybrid index (HI) for all 189 genetically sampled snakes (including juveniles; 41 Mojave, 60 hybrid, 88 Prairie). Each column is an individual snake, and colors signify the estimated proportion of ancestry from Mojave Rattlesnake (*Crotalus scutulatus*, blue) and Prairie Rattlesnake (*C. viridis*, red) genomes. The dashed horizontal lines indicate the cut off used for classifying hybrid individuals (0.05 and 0.95). The solid vertical lines indicate genetic group classifications.

**
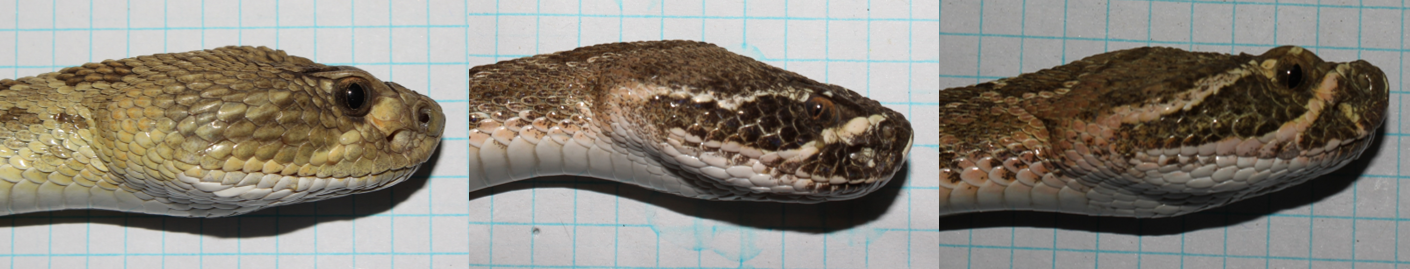
**

**Figure S.5:** Examples of “typical” snakes belonging to each genetic group. Left = *Crotalus scutulatus*, middle = *C.* *scutulatus* × *viridis*, right = *C. viridis*.

**Table S.1:** Alternative results of the relationship between the genetic group of individuals and various defensive and exploratory behaviors. These results are from the alternate dataset omitting the six snakes for which we were unable to estimate hybrid index. The pattern of statistically significant relationships does not differ from results presented in the main body of manuscript.

| **Factor of Interest** | **Best Fitting Models’ Predictor Variables** | **Test Statistic of Best and Simplest Model** | **Degree of Freedom** | **P-Value** |
| --- | --- | --- | --- | --- |
| **Rattle Behavior** | | | | |
| Means | Group + Age;  Group * Age | Group: X^2^ = 22.3 | 2 | < 0.001 |
|  |  | Age: X^2^ = 7.52 | 1 | 0.006 |
| **Time Spent in a Hidebox** | | | | |
| Means | Group + Age | Group: X^2^ = 0.587  Age: X^2^ = 4.87 | 2  1 | 0.746  0.027 |
| Variance of the Genetic Groups | NA | F = 0.141 | 2, 175 | 0.868 |
| **Quadrant Transitions** | | | | |
| Means | Group | Group: X^2^ = 1.99 | 2 | 0.369 |
| Variance of the Genetic Groups | NA | F = 0.806 | 2, 175 | 0.448 |
| **Time Spent Motionless** | | | | |
| Means | Group | Group: X^2^ = 0.309 | 2 | 0.857 |
| Variance of the Genetic Groups | NA | F = 0.749 | 2, 175 | 0.475 |
| **Strike Behavior** | | | | |
| Means | Group | Group: X^2^ = 2.52 | 2 | 0.283 |

**Table S.2:** Alternative results of the relationship between the hybrid index (HI) of hybrid snakes to various defensive and exploratory behaviors. These results are from the alternate dataset omitting the six snakes for which we were unable to estimate hybrid index. The pattern of statistically significant relationships does not differ from results presented in the main body of manuscript.

| **Best Fitting Models’ Predictor Variables** | **Test Statistic of Best and Simplest Model** | **Degree of Freedom** | **P-Value** |
| --- | --- | --- | --- |
| **Rattle Behavior** | | | |
| HI; HI + Age | HI: X^2^ = 5.45 | 1 | 0.020 |
| **Time Spent in a Hidebox** | | | |
| HI; HI + Age; HI * Age | HI: X^2^ = 0.417 | 1 | 0.518 |
| **Quadrant Transitions** | | | |
| HI * Age; HI * Age + Sex | HI: X^2^ = 0.760 | 1 | 0.383 |
|  | Age: X^2^ = 0.102 | 1 | 0.750 |
|  | Interaction: X^2^ = 7.78 | 1 | 0.005 |
| **Time Spent Motionless** | | | |
| HI; HI + Age; HI * Age; HI * Age + Sex | HI: X^2^ = 0.584 | 1 | 0.445 |
| **Strike Behavior** | | | |
| HI | HI: X^2^ = 0.190 | 1 | 0.663 |

**Table S.3:** Alternative results of the behavioral syndromes within each group between rattlesnakes’ defensive and exploratory behaviors. These results are from the alternate dataset omitting the six snakes for which we were unable to estimate hybrid index. The pattern of statistically significant relationships does not differ from results presented in the main body of manuscript. Boldened rows signify the existence of a significant behavioral syndrome identified with binomial Generalized Linear Models.

| **Defensive Behavior** | **Exploratory Behavior** | **X^2^** | **P-Value** | **Syndrome Relationship** | |
| --- | --- | --- | --- | --- | --- |
|  |  |  |  | **Defensiveness** | **Explorativeness** |
| *Crotalus scutulatus* | | | | | |
| Rattling (Adults) | Time in a hidebox | 2.09 | 0.148 | — | — |
|  | Time spent motionless | 2.35 | 0.125 | — | — |
|  | Quadrant transitions | 0.626 | 0.429 | — | — |
| Striking | Time in a hidebox | 3.24 | 0.072 | — | — |
|  | **Time spent motionless** | **4.29** | **0.038** | **↑** | **↓** |
|  | **Quadrant transitions** | **8.35** | **0.004** | **↑** | **↓** |
| *Crotalus viridis* | | | | | |
| Rattling (Adults) | Time in a hidebox | 0.0002 | 0.986 | — | — |
|  | Time spent motionless | 0.0002 | 0.988 | — | — |
|  | Quadrant transitions | 2.45 | 0.118 | — | — |
| Rattling (Juveniles) | Time in a hidebox | 2.01 | 0.157 | — | — |
|  | Time spent motionless | 2.18 | 0.140 | — | — |
|  | Quadrant transitions | 2.62 | 0.105 | — | — |
| Striking | **Time in a hidebox** | **5.86** | **0.015** | ↓ | ↓ |
|  | **Time spent motionless** | **6.14** | **0.013** | ↓ | ↓ |
|  | **Quadrant transitions** | **5.50** | **0.019** | ↓ | ↑ |
| *Crotalus scutulatus* × *viridis* | | | | | |
| Rattling (Adults) | Time in a hidebox | 0.425 | 0.514 | — | — |
|  | **Time spent motionless** | **6.22** | **0.013** | ↓ | ↓ |
|  | **Quadrant transitions** | **9.28** | **0.002** | ↓ | ↑ |
| Rattling (Juveniles) | Time in a hidebox | 0.475 | 0.491 | — | — |
|  | Time spent motionless | 0.898 | 0.343 | — | — |
|  | Quadrant transitions | 2.02 | 0.156 | — | — |
| Striking | Time in a hidebox | 0.009 | 0.924 | — | — |
|  | Time spent motionless | 0.482 | 0.488 | — | — |
|  | Quadrant transitions | 0.530 | 0.467 | — | — |
